# Metallic Lead Formation in Perovskites: Mechanisms, Suppression, and Future Directions

**DOI:** 10.1021/acsnano.5c11320

**Published:** 2025-09-26

**Authors:** Ahmed L. Abdelhady, Shakil N. Afraj, Yuki Haruta, Mohammed Misbah Uddin, Makhsud I. Saidaminov

**Affiliations:** † Department of Chemistry, 105955 Khalifa University, Abu Dhabi 127788, UAE; ‡ Center for Catalysis and Separation (CeCaS), 105955 Khalifa University, Abu Dhabi 127788, UAE; § Department of Chemistry, 8205 University of Victoria, 3800 Finnerty Road, Victoria, British Columbia V8P 5C2, Canada

**Keywords:** halide perovskite, metallic lead, Pb^0^, degradation, reversibility, suppression, mechanism, optoelectronics

## Abstract

Lead halide perovskites APbX_3_ (A = methylammonium, formamidinium, cesium; X = halogen) have advanced the field of optoelectronics, particularly in solar cells, photodetectors, and light-emitting diodes, due to their outstanding properties. However, a significant challenge remains unresolved: the formation of metallic lead (Pb^0^), which introduces deep-level defects that trap charge carriers and degrade device performance. The formation of Pb^0^ in perovskites can occur after their synthesis under various conditions, including high-energy radiation, light, heat, and moisture, and has also been observed during perovskite crystallization. Thus, it is crucial to understand the underlying mechanisms of Pb^0^ formation and its suppression pathways. Recent studies have explored various strategies to suppress Pb^0^ formation, including compositional engineering, additive incorporation, and protective passivation layers. In this review, we discuss the origins of Pb^0^ formation in perovskites, focusing on the mechanisms driving this process under different environmental conditions, and then strategies for suppressing Pb^0^ formation, including compositional engineering and passivation techniques. By addressing these aspects, we seek to identify pathways for enhancing the stability and performance of perovskite-based devices, enabling their widespread adoption in commercial applications.

## Graphical abstract

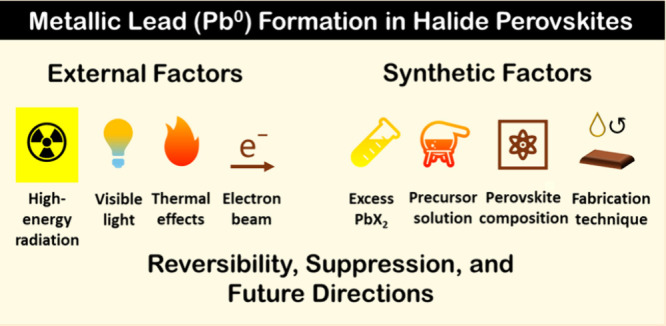

## Introduction

Lead halide perovskites APbX_3_ (A = methylammonium, formamidinium, cesium; X = halogen ion) have attracted significant attention in the field of optoelectronics due to their superior optical and electronic properties.^1,2^ However, the formation of metallic lead (Pb^0^) within the perovskite structure hinders the commercialization and weakens the long-term stability of perovskite-based devices.^3–5^ This is because Pb^0^ forms deep defects that act as traps, which quenches the excitons. Furthermore, Pb^0^ promotes nonradiative recombination, impairs charge transport, and destabilizes interfacial energetics. Studies have shown that Pb^0^ accumulation degrades the interfaces between the perovskite and charge transport layers, leading to increased ion migration and hence severe hysteresis.^3,6,7^ Pb^0^ usually forms together with neutral iodine (I^0^), especially under illumination or heat, accelerating degradation through redox interactions. Wang et al.^4^ demonstrated that a Eu^3+^/Eu^2+^ redox shuttle can simultaneously oxidize Pb^0^ and reduce I^0^, prolonging device lifetimes to over 1,500 h under thermal or light stress. Pb^0^ also induces structural and optical instability, particularly in perovskite nanocrystals, where its presence quenches light emission, broadens spectral features and contributes to photoinstability, which is a critical concern for light-emitting diode (LED) applications.^5^ From a structural perspective, Pb^0^ formation disrupts the lattice, promotes the loss of halide ions, and destabilizes grain boundaries, especially under ambient fabrication conditions.^8,9^ Together, these findings provide evidence that links Pb^0^ to multiple failure pathways across perovskite solar cells, LEDs, and detectors. Therefore, mitigating Pb^0^ formation is not only about reducing efficiency losses but also ensuring the commercial viability of perovskite optoelectronic devices. This review systematically examines the environmental, chemical, and synthetic factors contributing to Pb^0^ generation and highlights state-of-the-art strategies from compositional tuning to additive engineering and surface passivation, to prevent its formation and enhance the device stability.

The Pb^0^ formation is primarily attributed to the decomposition of the perovskite material caused by various external influences, such as exposure to light, heat, moisture, and high-energy radiation (e.g., X-rays), among others.^10,11^ Additionally, Pb^0^ is formed during the crystallization and growth of the perovskite material.^4,12^ In the coming sections, we discuss the influence of external factors, synthetic factors, and device architecture on the formation of Pb^0^. We also explore the reversibility of this process and identify possible passivation techniques. At the end of each section, we provide a perspective/outlook that could guide in understanding the mechanisms and developing effective strategies to enhance the stability and performance of perovskite-based devices.

## Early Discoveries of Pb^0^ in Halide Perovskites

The photodecomposition of PbI_2_ into Pb^0^ and I_2_ gas has been observed in 1965, long before the booming of the halide perovskite field.^13^ Similarly, the photolysis of triiodoplumbate­(II) complexes (PbI_3_^–^) into Pb^0^ was also reported.^14^ The first mention of detected Pb^0^ in halide perovskites was reported in 2014.^15^ Using X-ray photoemission spectroscopy (XPS), a signal corresponding to Pb^0^ in MAPbI_3_ films was detected (Figure 1a), which was suggested to be the reason for the Fermi level pinning close to the conduction band, i.e. making the perovskite film highly n-type. This was followed by several other reports in the same year that similarly detected Pb^0^ in the perovskite material.^16–18^ A hypothesis for the formation of Pb^0^ was suggested to be linked to the removal of I_2_ from MAPbI_3_.^16^ This was based on observing that the presence of Pb^0^ is associated with understoichiometry in iodine. Interestingly, the Pb^0^ signal in XPS measurements was notable for the two-step spin-coated MAPbI_3_ films, whereas it was vaguely seen for the one-step spin-coated films (Figure 1b). This suggests that Pb^0^ formation is not solely due to X-ray irradiation during the XPS measurements; the synthesis technique could also be a factor. On the other hand, it was also proposed that upon irradiation of the perovskite material with a 355 nm laser, electrons are trapped at Pb^2+^ centers, forming diamagnetic Pb clusters that also serve as color centers.^17^ These centers are defects within the crystal lattice that trap electrons, resulting in the absorption of extended wavelengths of light and giving a darker color to the material. Similar to the first report in 2014,^15^ the following work reported that the Fermi level for MAPbI_3_ films deposited on n-type substrates is at or slightly above the conduction band minima (CBM), which was correlated with the detection of Pb^0^ signals in the XPS measurements.^18^ The work provided two possible explanations for the formation of Pb^0^, which are either due to the decomposition of unreacted PbI_2_ precursor directly or the decomposition of the perovskite into its precursors (MAI and PbI_2_) followed by the decomposition of PbI_2_ to Pb^0^ and I_2_ as demonstrated in the equations below:

$${\mathrm{MAPbI}}_{3}\to \mathrm{MAI}+{\mathrm{PbI}}_{2}$$

$${\mathrm{PbI}}_{2}\to {\mathrm{Pb}}^{0}+I_{2}$$

**Figure 1:**
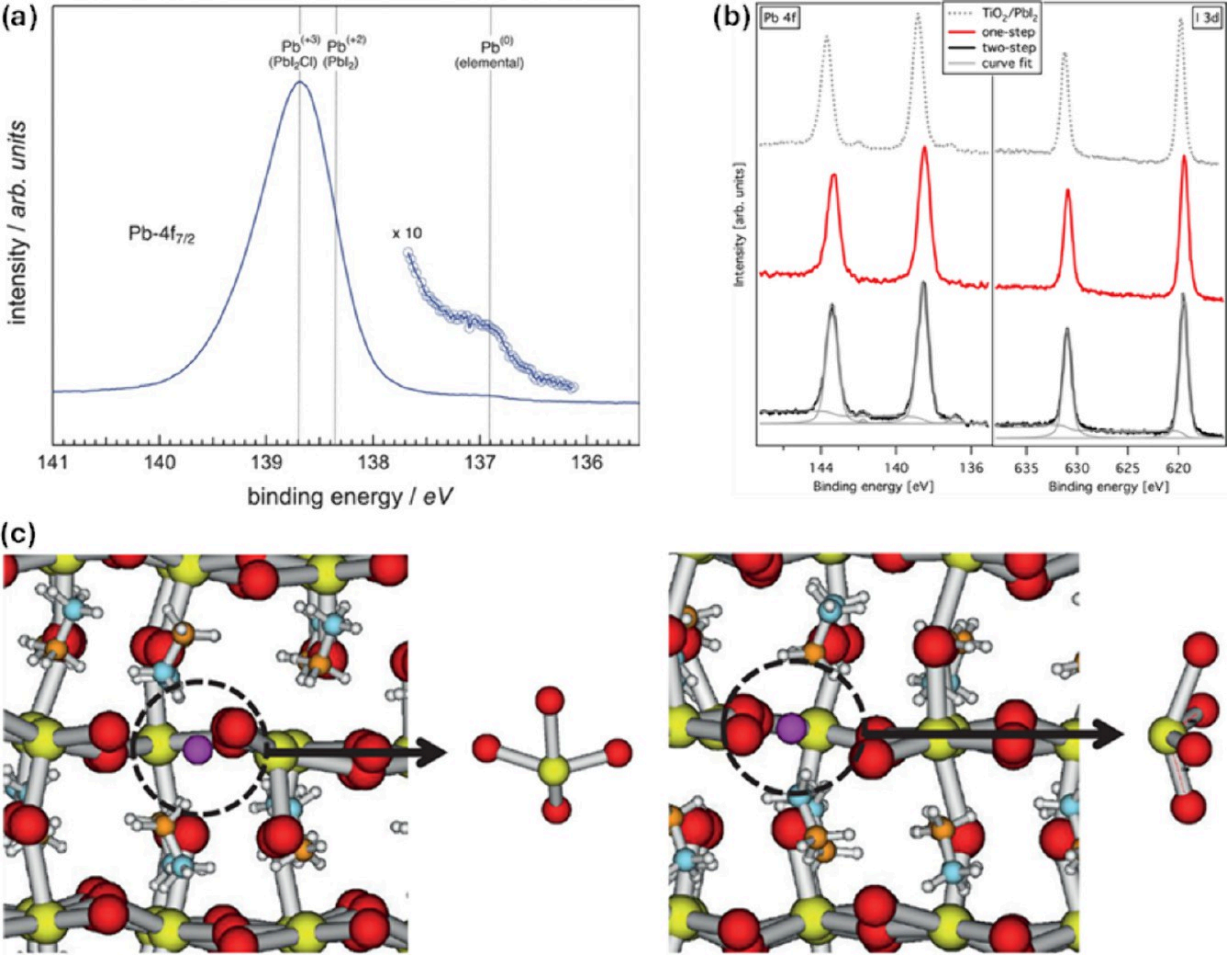
(a) High-resolution Pb 4f_7/2_ core-level spectrum recorded from a MAPbI_3_ film. Reproduced with permission from ref 15. Copyright 2013 Wiley-VCH. (b) Core-level spectra of Pb 4f and I 3d for PbI_2_ and MAPbI_3_ films prepared via one- and two-step spin-coating methods. Reproduced from ref 16. Copyright 2014 American Chemical Society. (c) Illustration of an undercoordinated Pb atom (shown in purple and highlighted by the dotted ring) at the two defect structures: structure 1 (left) and structure 2 (right). The enlarged inset highlights the local coordination environment. Reproduced from ref 19. Copyright 2014 American Chemical Society.

Through density functional theory (DFT) calculations on two simulated MAPbI_3_ structures with undercoordinated Pb^2+^ (Figure 1c), Pb^0^ was concluded to form through grouping of these undercoordinated Pb^2+^ forming larger Pb^0^ clusters.^19^ This confirms the previously proposed theory that the formation of Pb^0^ is due to undercoordinated Pb^2+^ through the loss of I_2_.^16^ These early discoveries regarding Pb^0^ formation stimulated detailed studies on the factors contributing to its formation and its formation mechanism.

## Influence of External Factors on Pb^0^ Formation

### High-Energy Radiation under Different Conditions

Several works have identified X-ray exposure as the primary origin of Pb^0^ formation in halide perovskites. The extent of X-ray damage and the pathway of Pb^0^ formation are affected by the sample fabrication conditions, the atmosphere and temperature under which the perovskite samples are being exposed to the X-rays, X-ray fluence levels, exposure duration, and the aging process. For instance, X-ray irradiation produced more Pb^0^ in spin-coated MAPbI_3–*x*_Cl_*x*_ films annealed in air than in those annealed under N_2_ (Figure 2a).^20^ Also, upon X-ray exposure, the Pb^0^ content in the perovskite films gradually increased, particularly when the samples were heated between 100 and 140 °C (Figure 2b).^21^ This was supported by XPS data showing decreased X/Pb and N/Pb ratios during X-ray exposure under heating. Notably, heating the perovskite samples without X-ray exposure resulted in degradation of the perovskite to only PbI_2_, emphasizing that X-ray exposure is the origin of Pb^0^ formation and heating facilitates it. Prolonged X-ray irradiation under high vacuum conditions also led to the formation of Pb^0^ in MAPbI_3_ films.^22^ Note that an ultrahigh vacuum (UHV) environment without exposure to X-ray irradiation did not lead to Pb^0^ formation, suggesting it was originated from the X-rays, not vacuum.^23^

**Figure 2:**
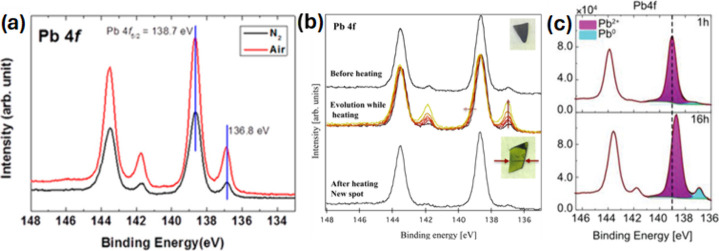
(a) Pb 4f core-level XPS spectra of MAPbI_3_ films annealed in air and in N_2_ atmosphere. Reproduced from ref 20. Copyright 2015 American Chemical Society. (b) Pb 4f core-level XPS spectra of MAPbI_3_/meso-TiO_2_/Si, before, during and after annealing. Reproduced from ref 21. Copyright 2015 American Chemical Society. (c) Pb 4f core-level XPS spectra of Cs_0.05_(MA_0.14_,FA_0.86_)_0.95_Pb­(I_0.84_,Br_0.16_)_3_ films recorded after 1 and 16 h of X-ray exposure at the same spot. Reproduced with permission from ref 24. Copyright 2023 Wiley-VCH.

The formation of Pb^0^ in MAPbI_3_ films due to X-ray irradiation is time-dependent with Pb^0^ fraction reaching 9% of the total Pb content after 16 h of X-ray exposure (Figure 2c).^24^ The X-ray intensity also impacts the Pb^0^ formation. At low X-ray fluence levels (∼10^13^ photons cm^–2^) for 1 h of exposure, the formation of Pb^0^ is minimal, primarily inducing bulk defects.^24^ In contrast, at higher fluence levels (∼10^14^ photons cm^–2^) for >8 h of exposure, surface recombination is increased especially within the central spot of X-ray exposure, leading to a more significant formation of Pb^0^. Additionally, long-term storage in air also affects the Pb^0^ formation in halide perovskites upon exposure to X-ray.^25^ It was suggested that the aging of the perovskite resulted in its decomposition to only PbI_2_, which, under XPS measurements, decomposed to Pb^0^ due to the X-rays.

Under X-ray exposure, the perovskite structure undergoes chemical changes, leading to the formation of Pb^0^. This occurs concurrently with the decomposition of MAPbI_3_ into PbI_2_, MAI, CH_3_NH_2_, and HI gases.^23^ The mechanism of Pb^0^ formation as a result of X-ray exposure involves the reduction of Pb^2+^ ions in the perovskite lattice to Pb^0^. This reduction process is likely initiated by the interaction of X-rays with the perovskite surface, leading to the degradation of the organic and iodine components and the formation of electronic defects that subsequently lead to the reduction of Pb^2+^ to Pb^0^.^24^

While the above works focused mainly on the iodide-based perovskites, the X-ray damage on MAPbBr_3_ was also investigated.^26^ MAPbBr_3_ single crystals (SCs) showed minimal Pb^0^ formation under vacuum in dark, but approximately 10% of the surface underwent degradation to Pb^0^ when exposed to X-rays. N_2_ environment delayed this X-ray degradation for 9 h under identical conditions, in agreement with the above-mentioned results on MAPbI_3–*x*_Cl_*x*_ films.^20^ However, after a longer X-ray exposure time (14 h) under the N_2_ atmosphere, a similar amount of Pb^0^ (10%) was recorded. The X-ray irradiation of MAPbBr_3_ SCs under O_2_ and H_2_O environments was also explored. O_2_ cannot provide surface protection against X-rays, as in the case of N_2_. Under the H_2_O environment, Pb^2+^ degraded to Pb^0^ upon X-ray exposure, reaching 17% after prolonged X-ray exposure due to the degradation of PbBr_2_ formed by the reaction between MAPbBr_3_ and water. However, the Pb^0^ content dropped subsequently due to the formation of Pb­(OH)_2_.

Gamma (γ) rays exposure similarly induces the formation of Pb^0^ in perovskite films.^27^ While the Pb^0^ diffraction peak in XRD was detected for the mixed A-site (CsFA)­PbI_3_ and (CsMAFA)­PbI_3_ perovskite films at a dose of 5 and 7 MGy of γ rays, respectively, the Pb^0^ diffraction peak started to evolve in the case of the MAPbI_3_ film at a lower dose of 1–2 MGy.

From the above studies, it is evident that exposure to high-energy radiation such as X-rays and γ rays is the primary driver for the reduction of Pb^2+^ to Pb^0^ through defect formation. These defects not only serve as electron traps, but also provide reactive sites that accelerate the transformation of Pb^2+^ into metallic Pb^0^. On the other hand, heating accelerates the decomposition and facilitates the reduction reaction, as elevated temperatures enhance ionic mobility and may destabilize the organic cation framework. Meanwhile, vacuum condition helps removing the volatile decomposition products, shifting the chemical equilibrium toward Pb^0^ formation. Understanding the exact mechanisms by which different environmental factors influence Pb^0^ formation under X-ray exposure will be essential in improving the stability and long-term stability of perovskite-based devices.

### Visible Light under Different Conditions

One of the main explored sources for the Pb^0^ formation is the exposure of the halide perovskites to visible light. Experimental studies have demonstrated that light-triggered processes, influenced by environmental factors such as vacuum, inert atmospheres, or air, drive structural and chemical changes in perovskite materials. For instance, Pb^0^ is formed in a vacuum under white light illumination, while lead salts (PbO, Pb­(OH)_2_, and PbCO_3_) are formed after a photoinduced degradation in air.^28^ Similar to the X-ray-induced damage,^20,26^ the photoinduced degradation is insignificant under an N_2_ environment. Control experiments in the absence of light confirmed that light irradiation is the primary trigger for the Pb^0^ formation, as in dark, MAPbI_3_ thin films exhibited only slow degradation under vacuum and negligible degradation in air, as evidenced by XRD measurements (Figure 3a). Furthermore, under vacuum/light conditions, in situ XRD measurements (Figure 3b) demonstrated that MAPbI_3_ initially (up to 6 h) decomposes into Pb^0^ and PbI_2_. Afterward, the Pb^0^ content increased gradually as PbI_2_ was photolyzed. It was also reported that PbI_2_ undergoes photodecomposition to Pb^0^ and I_2_ gas under illumination with light with the wavelengths shorter than 530 nm.^29^ On the other hand, MAPbI_3_ exhibited no wavelength threshold under which photodecomposition takes place. Under illumination, the degradation of MAPbI_3_ takes place as illustrated in the equation below:

$${\mathrm{MAPbI}}_{3}\left(s\right)\overset{h\nu }{\to }{\mathrm{PbI}}_{2}\left(s\right)+{\mathrm{Pb}}^{0}\left(s\right)+I_{2}\left(g\right)+{\mathrm{CH}}_{3}{\mathrm{NH}}_{2}\left(g\right)+{\mathrm{CH}}_{3}I\left(g\right)+\mathrm{HI}\left(g\right)+{\mathrm{NH}}_{3}\left(g\right)$$

**Figure 3:**
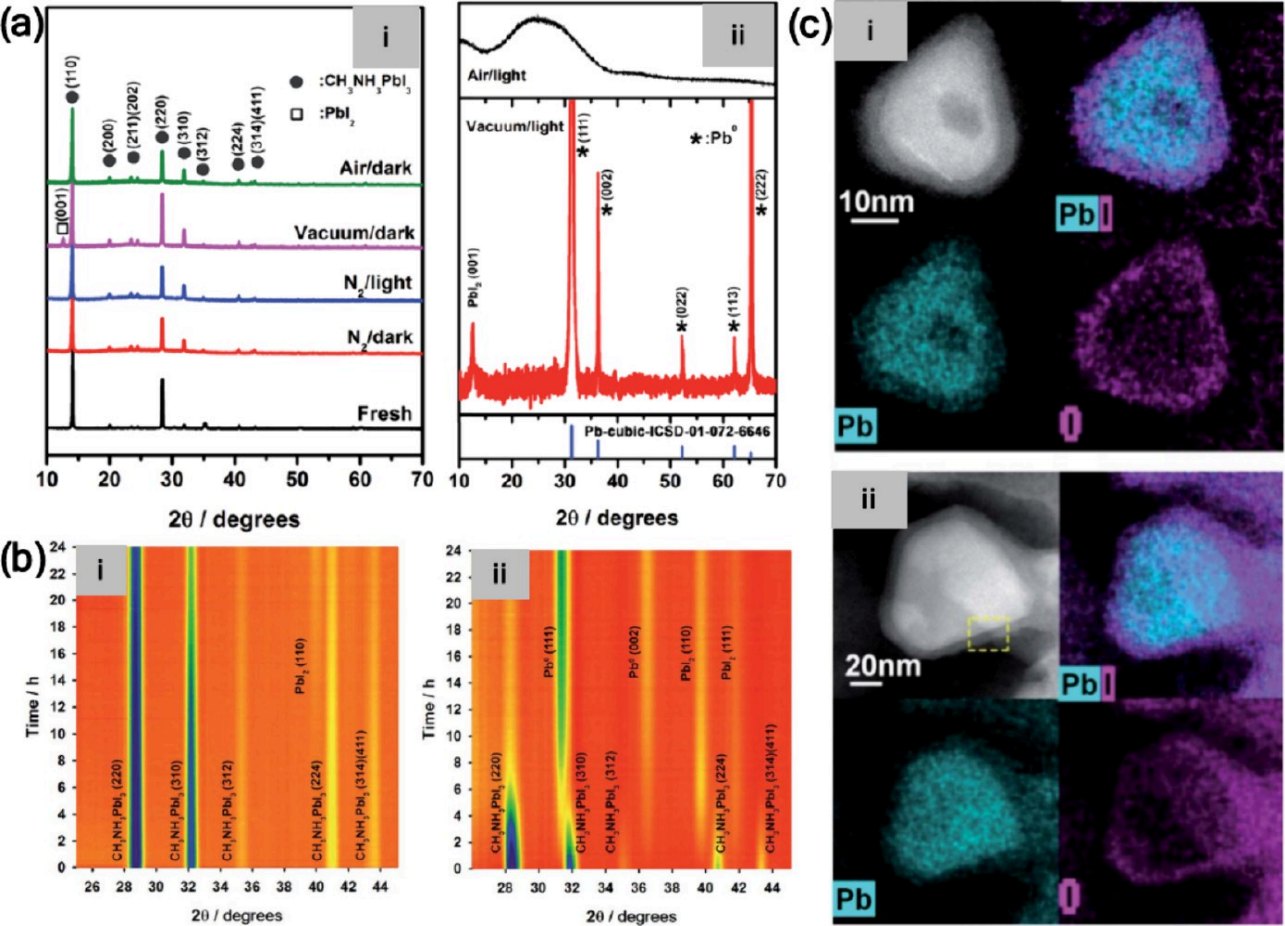
(a) X-ray diffraction patterns of MAPbI_3_ films degraded for 24 h in various atmospheres under (i) dark conditions and (ii) light conditions. Reproduced with permission from ref 28. Copyright 2016 Royal Society of Chemistry. (b) In situ XRD of MAPbI_3_ films at 350 K under (i) vacuum/dark and (ii) vacuum/light, along with the integrated intensity evolution under vacuum/light. Reproduced with permission from ref 28. Copyright 2016 Royal Society of Chemistry. (c) HAADF images and corresponding EDS mappings showing the Pb@PbI_2–*x*_ core/shell structure. Reproduced with permission from ref 36. Copyright 2021 Royal Society of Chemistry.

Recently, it was demonstrated that the formation of Pb^0^ in FAPbI_3_ perovskite is triggered by exposure to white light rather than X-rays.^30^ White light encompasses a broad spectrum of wavelengths, including visible and near-infrared light, which the perovskite material can readily absorb. In contrast, X-rays typically have higher energies and penetrate deeper into the material without being fully absorbed. Therefore, the energy absorbed from white light may be more effective in initiating Pb^0^ formation within the perovskite lattice compared to X-rays. Light exposure, particularly within the absorption range of PbI_2_ domains, accelerates the degradation.

Degradation of mixed halide perovskites, namely, MAPbI_3–*x*_Cl_*x*_ and MAPbBr_3–*x*_Cl_*x*_ thin films under UHV by white light illumination, revealed that Pb^0^ was recorded only in the case of MAPbI_3–*x*_Cl_*x*_, while the decomposition product of MAPbBr_3–*x*_Cl_*x*_ was PbBr_2–*x*_Cl_*x*_.^31^ This suggests that PbI_2_ is photosensitive while PbBr_2–*x*_Cl_*x*_ is not. The effect of white light illumination on MAPbI_3–*x*_Cl_*x*_ thin films showed that illumination increased the Pb^0^ content leading to strong Fermi level pinning.^32^ Angle-dependent XPS measurements on the MAPbI_3–*x*_Cl_*x*_ thin films, revealed that Pb^0^ is predominantly at the surface level. Under white light illumination, mixed perovskite compositions (FA_0.83_MA_0.17_Pb­(I_0.83_Br_0.17_)_3_, and Cs_0.1_(FA_0.83_MA_0.17_)_0.9_Pb­(I_0.83_Br_0.17_)_3_) showed slower degradation and lower Pb^0^ content compared to the single cation composition (MAPb­(I_0.83_Br_0.17_)_3_).^33^ Importantly, the degradation of all three mixed perovskite compositions was confirmed to be photoinduced rather than being due to the UHV condition; in dark UHV conditions, even the single cation composition showed no sign of Pb^0^ after 72 h.

In terms of devices, the light-induced degradation in mixed cation/halide perovskite solar cells (PSCs) under 1 sun illumination revealed that Pb^0^ was observed near the cathode interface.^34^ This reduction is facilitated by the photogenerated electrons and holes generated during solar cell operation. Furthermore, the formation of Pb^0^ is promoted through (i) exposure to light and O_2_ and (ii) high interfacial or grain boundaries defect density. Notably, the Pb^0^ formation is not caused by X-ray damage, as the Pb^0^ XPS signal did not increase upon repeated measurements in the same position.

The mechanism of the photoinduced degradation process under vacuum was proposed to take place through forming the highly volatile MAI compound, resulting in the creation of iodine vacancies (V_I^–^_), which are capable of capturing photogenerated electrons.^28^ It was suggested that the trapped electron initiates the reduction of neighboring Pb^2+^ to Pb^+^, and, owing to the thermodynamic instability of Pb^+^, two Pb^+^ ions subsequently undergo further reaction, resulting in the formation of Pb^0^ and Pb^2+^.^28^ The formation of Pb^0^ leads to vacancies within the MAPbI_3_ lattice, facilitating the trapping of photogenerated holes, which, in turn, oxidize I^–^ to I_2_. Similar results were also proposed by others.^35^ The overall reaction is illustrated in the equations below:

$$2{\mathrm{MAPbI}}_{3}\overset{\mathrm{vacuum/light}}{\to }2\mathrm{MA}+2\mathrm{HI}+{\mathrm{PbI}}_{2}+I_{2}$$

$${\mathrm{PbI}}_{2}\overset{\mathrm{vacuum/light}}{\to }\mathrm{Pb}+I_{2}$$

Under Ar atmosphere and white light illumination, the formation mechanism of Pb^0^ in MAPbI_3_ was shown to proceed starting at the grain boundaries.^36^ The low migration energy of I^–^ ions prompts the formation of iodide deficiencies at grain boundaries. Consequently, photoexcited electrons localize at the grain boundaries, reducing Pb^2+^ to Pb^0^. Simultaneously, the photoexcited holes oxidize migrated I^–^ ions into I_2_. This is followed by forming an intermediate amorphous PbI_2–*x*_ phase on Pb^0^ clusters, forming a core–shell structure of Pb@PbI_2–*x*_ that was confirmed by atomic resolution HAADF and EDS mappings (Figure 3c).

The degradation of MAPbI_3_ thin films under laser irradiation (408 nm and illumination intensity of 6.8 × 10^–3^ W/mm^2^) under UHV has also been observed, where it was found that the PbI_2_ liberated from MAPbI_3_ undergoes additional decomposition into Pb^0^ and iodine solely through irradiation.^37^ Moreover, it was confirmed that the degradation is only photoinduced rather than being thermally driven due to heating by the laser beam. Nevertheless, it was suggested that for thicker samples, heating by laser beam could play a more significant role. Recently, it was shown that MAPbI_3_ films, when exposed to a continuous-wave 450 nm laser, will form both iodide vacancies and Pb^3+^ centers.^38^ These defects will trap charge carriers, leading to the formation of Pb^0^. In this study, the authors detected the presence of Pb^3+^ and Pb^0^ using ^207^Pb nuclear magnetic resonance (NMR) and electron paramagnetic resonance (EPR) methods, and they concluded that there are around 40 Pb^0^ atoms per Pb^3+^ defect in the film.

Researchers also explored the impact of visible laser (405, 515, and 532 nm) illumination on mixed Br/I perovskites.^39–41^ The presence of Pb^0^ is observed when the laser is turned on, and its concentration increases proportionally with the intensity of the laser. Under illumination, the mixed halide perovskites underwent phase segregation, forming Br-rich and I-rich domains as illustrated in Figure 4a,b. The charge carriers preferentially localize in the I-rich regions due to its narrower bandgap, causing the formation of trap states. The formation of Pb^0^ in the I-rich domain was indicated by increasing Pb^0^/Pb_total_ ratios as confirmed by XPS analysis. Formation of Pb^0^ was attributed to redox chemistry involving Pb^2+^ and Pb^0^ species, along with I^–^ and I_3_^–^ ions. Under continuous light illumination, Pb^0^ and I_3_^–^ defects accumulate at the grain boundaries, increasing the energy of the crystal lattice and promoting their migration to the surface (Figure 4c). The presence of multiple surface defects, such as dangling bonds or undercoordinated Pb^2+^ ions, may accelerate the accumulation of these defects at grain boundaries. Furthermore, grain boundaries have been shown to be more photoactive than the grains themselves, contributing to the preferential accumulation of Pb^0^ and I_3_^–^ species.

**Figure 4:**
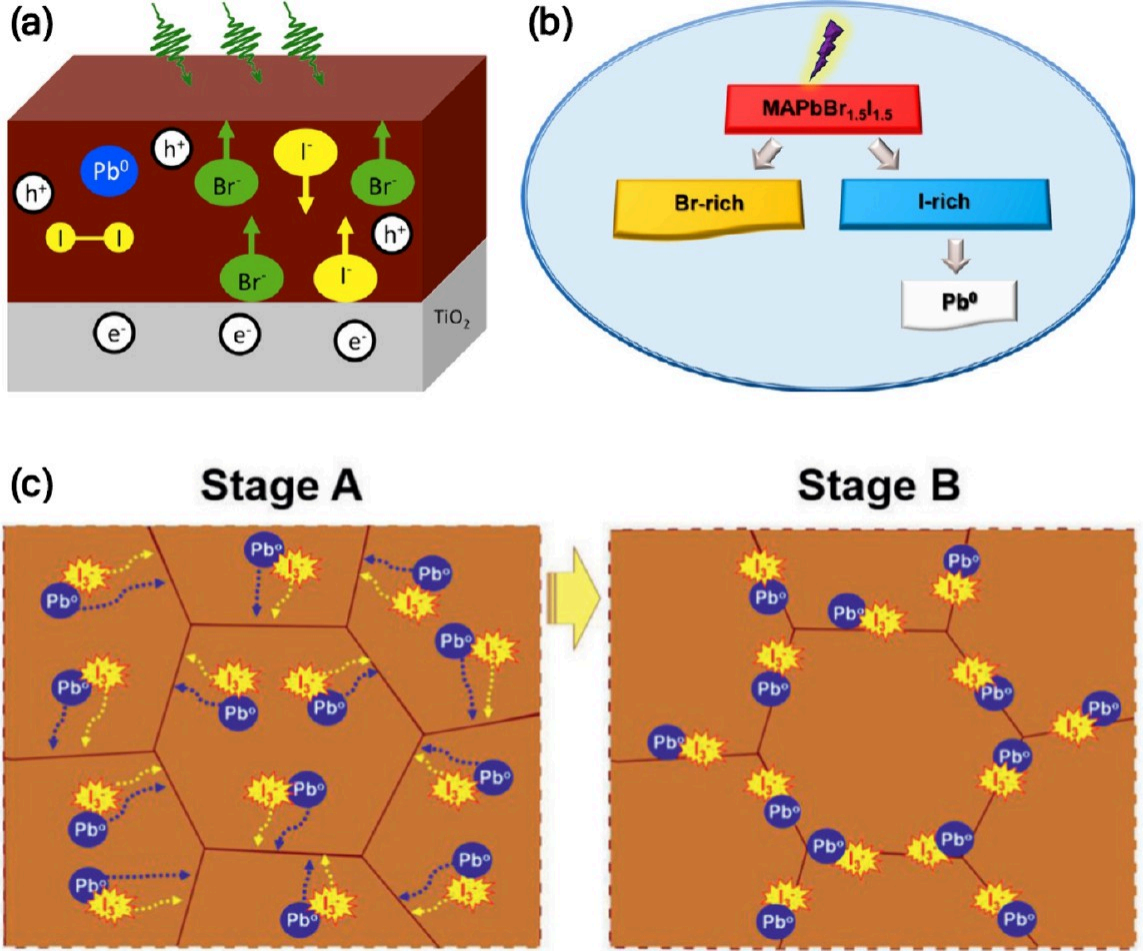
(a) Schematic illustration showing composition changes in a perovskite film under illumination, where Pb^0^ and I_2_ are formed and the surface becomes more halide-rich with Br^–^ ions migrating to the surface. Reproduced from ref 39. Copyright 2017 American Chemical Society. (b) Schematic illustration of the irreversible transformation of a mixed halide perovskite upon light illumination. Reproduced from ref 40. Copyright 2018 American Chemical Society. (c) Schematic illustration of the proposed pathway for the light-induced halide phase segregation in mixed-halide perovskites. Stage A: photochemical generation of Pb^0^ and I_3_^–^ defect. Stage B: migration of the defects toward the grain boundaries. Reproduced with permission from ref 41. Copyright 2021 Wiley-VCH.

Others have also recorded Pb^0^ in quasi-2D perovskites, namely PEA_2_FA_2_Pb_3_Br_10_ (PEA = phenylethylammonium).^42^ Similarly, they attributed the origin of Pb^0^ to the photolysis of PbX_2_ under photoexcitation (365 nm), leading to the release of halide ions and the formation of Pb^0^. The authors have also recorded that the formation of Pb^0^ is associated with the diminishing of the quasi-2D diffraction peaks and along with increased intensity of the 3D perovskite peaks.

The stability of MAPbI_3_ under different conditions indicated that the formation of Pb^0^ is due to combined photodegradation and thermal effects.^43^ In a N_2_ atmosphere, the light of a mercury lamp degrades the perovskite into a mixture of PbI_2_ and Pb^0^ when thermally aged at 75 °C. In contrast, films annealed (85–95 °C) in the dark or aged with light and oxygen at room temperature resulted in the formation of only PbI_2_. Interestingly, the results also show that the N_2_ atmosphere does not necessarily provide passivation to the perovskite as was previously suggested.^28^

In summary, the understanding of Pb^0^ formation due to visible light illumination in perovskite materials, particularly MAPbI_3_, has advanced considerably with recent studies. Under white LED illumination, MAPbI_3_ films degrade from Pb^2+^ to Pb^0^ through an intermediate stage of PbI_2_. This process is accelerated under UHV conditions and is attributed to the creation of iodine vacancies, which capture photogenerated electrons and results in the reduction of Pb^2+^ to Pb^0^. Additionally, laser irradiation has been shown to decompose PbI_2_ into Pb^0^, highlighting the influence of specific wavelengths on degradation. FAPbI_3_ also shows a similar decomposition trend but possibly with a different degradation rate due to its larger cation size and stronger hydrogen bonding, which may confer enhanced lattice stability. In the case of the mixed Br/I perovskites, the formation of Pb^0^ is a result of the phase segregation to Br-rich and I-rich regions followed by the degradation of the I-rich region into Pb^0^. The light-induced degradation under different conditions, including the presence of O_2_, N_2_, and the intensity of the laser, significantly impacts the extent of Pb^0^ formation. For instance, oxygen may facilitate the degradation process through superoxide formation, while high-intensity, pulsed lasers can induce localized heating or structural changes.

The fact that Pb^0^ forms more easily under specific illumination conditions, such as laser light, compared to thermal annealing or X-ray irradiation, indicates that light-induced degradation is particularly significant for the operational stability of perovskite materials. This highlights the need to optimize light exposure conditions and develop strategies to mitigate photoinduced degradation, especially in applications involving high-intensity light or prolonged illumination. Moreover, the different degradation behavior observed in mixed cation perovskites compared to single cation perovskites suggests that tailoring the composition can impact the stability and degradation pathways. Future research should focus on exploring these variations in more detail and developing materials that can endure operating conditions in real-life optoelectronic applications. However, compositional engineering must balance other trade-offs, such as charge transport, crystallinity, and bandgap tunability.

### Thermal Effects

Another external factor that can cause the Pb^0^ formation is thermal annealing in ambient atmosphere.^44^ XPS measurements (Figure 5a) under different atmospheres on MAPbI_3_ thin films showed no Pb^0^ signal for the fresh sample. However, after annealing at 85 °C in air and in an O_2_- and H_2_O-free environment, the Pb^0^ signal evolved, though at a much slower rate for the latter. The products of the thermal degradation of MAPbI_3_ under vacuum and under O_2_ or H_2_O atmosphere showed to be different.^45^ Initially, the perovskite degraded completely into PbI_2_ when the temperature reached 150 °C. However, between 200 and 300 °C, PbI_2_ degrades with the final product being Pb^0^ under vacuum and Pb oxides and hydroxides under O_2_ or H_2_O atmosphere. These conclusions were based on the N:Pb ratio for the first step, where it dropped to 0 at 150 °C, indicating the release of all MA and formation of PbI_2_. The I:Pb ratio was used for the second step, which indicated the formation of Pb^0^ under vacuum as the ratio dropped to 0 at 300 °C. Meanwhile, under O_2_ or H_2_O atmosphere, the ratio decreased to 0.6 and 0.3, respectively, indicating the formation of other Pb compounds. The I:Pb and N:Pb, and Pb^0^% at different annealing temperatures are all displayed in Figure 5b.

**Figure 5:**
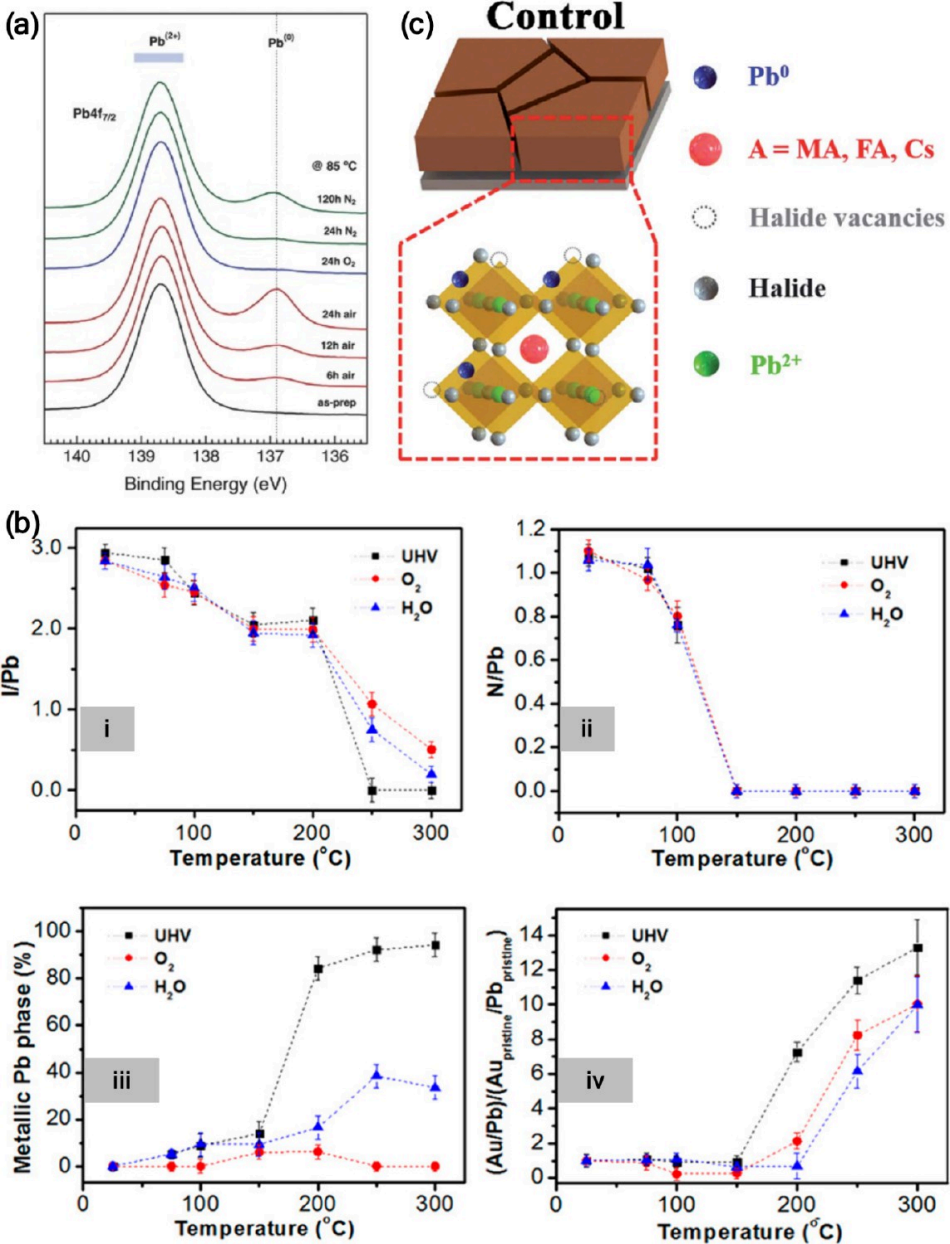
(a) XPS spectra of Pb 4f_7/2_ acquired from degraded ITO/TiO_2_/perovskite samples exposed to 85 °C for up to 24 h in different atmospheric conditions. Reproduced with permission from ref 44. Copyright 2015 Wiley-VCH. (b) Atomic ratios of (i) I/Pb, (ii) N/Pb, (iii) Pb^0^/Pb_total_, and (iv) Au/Pb_total_ for the MAPbI_3_ films as a function of annealing temperature under vacuum, and under 1 mbar pressure of O_2_ and H_2_O. Reproduced from ref 45. Copyright 2017 American Chemical Society. (c) Schematic illustration showing a Pb^0^ cluster. Reproduced with permission from ref 46. Copyright 2021 Royal Society of Chemistry.

In perovskites involving organic cation(s) in the A-site, the origin of Pb^0^ formation was attributed to the evaporation of the organic components from the perovskite surface and the creation of halide vacancies during thermal annealing processes as illustrated in Figure 5c.^46,47^ When perovskite films undergo thermal stress during fabrication or operation, organic cations may decompose, releasing species that react with Pb^2+^ in the perovskite structure, leading to the formation of Pb^0^ clusters. This process is facilitated by the soft Lewis acid–base reaction between Pb^2+^ and alkylamines produced by the thermal decomposition of organic cations.

Thermal exposure plays a dual role: on one hand, it catalyzes desirable crystallization and defect healing during fabrication; on the other, it accelerates the loss of volatile organic cations. The formation of Pb^0^ under heat is directly linked to the decomposition of MA and FA, which produce reactive byproducts (e.g., CH_3_NH_2_, NH_3_, HI) that disrupt lattice stability. An important insight from the above studies is the need for controlled environmental conditions during the fabrication and operation of the perovskite-based devices, as evidenced by the stark differences in the degradation pathways and Pb^0^ formation under vacuum versus in air. The crucial role of the organic cation thermal decomposition in the formation of Pb^0^ should be further explored, along with the investigation of photodegradation under thermal effects, which is needed for comprehensive stability testing across different environmental and operational conditions. An important future direction could involve mapping the activation energies of various degradation steps under different atmospheric conditions to predict device longevity. Further, improving thermal robustness could be achieved through a shift toward all-inorganic or hybrid systems with thermally resilient organic cations.

### Electron Beam

To explore the roles of electron beam and vacuum on perovskite degradation, perovskite films were fabricated through sequential sublimation of PbI_2_ and MAI directly on a Cu carbon-coated grid that was then loaded into the vacuum chamber of the TEM.^48^ The degradation of the perovskite layer was reported to occur due to the perturbation of the perovskite structure by the electron irradiation (even at a low dose), resulting in the release of volatile species and the aggregation of Pb-related defects at the boundaries of MAPbI_3_ grains in the form of Pb-clusters. Two competing transformation pathways were observed: MAPbI_3_ to Pb and MAPbI_3_ to PbI_2_. The degradation of the grain core to PbI_2_ was evidenced to prevent further evolution of the Pb-clusters. The TEM images and schematic of Pb^0^ clusters at the grain boundaries are displayed in Figure 6. In a recent study,^49^ it was suggested that the electron beam (in TEM) results in a strong electric field, which derives the Br^–^ ions to migrate. This leads to the depletion of the Br^–^ ions from the periphery region and as a result the Pb^0^ is formed in these regions and not at the beam center.

**Figure 6:**
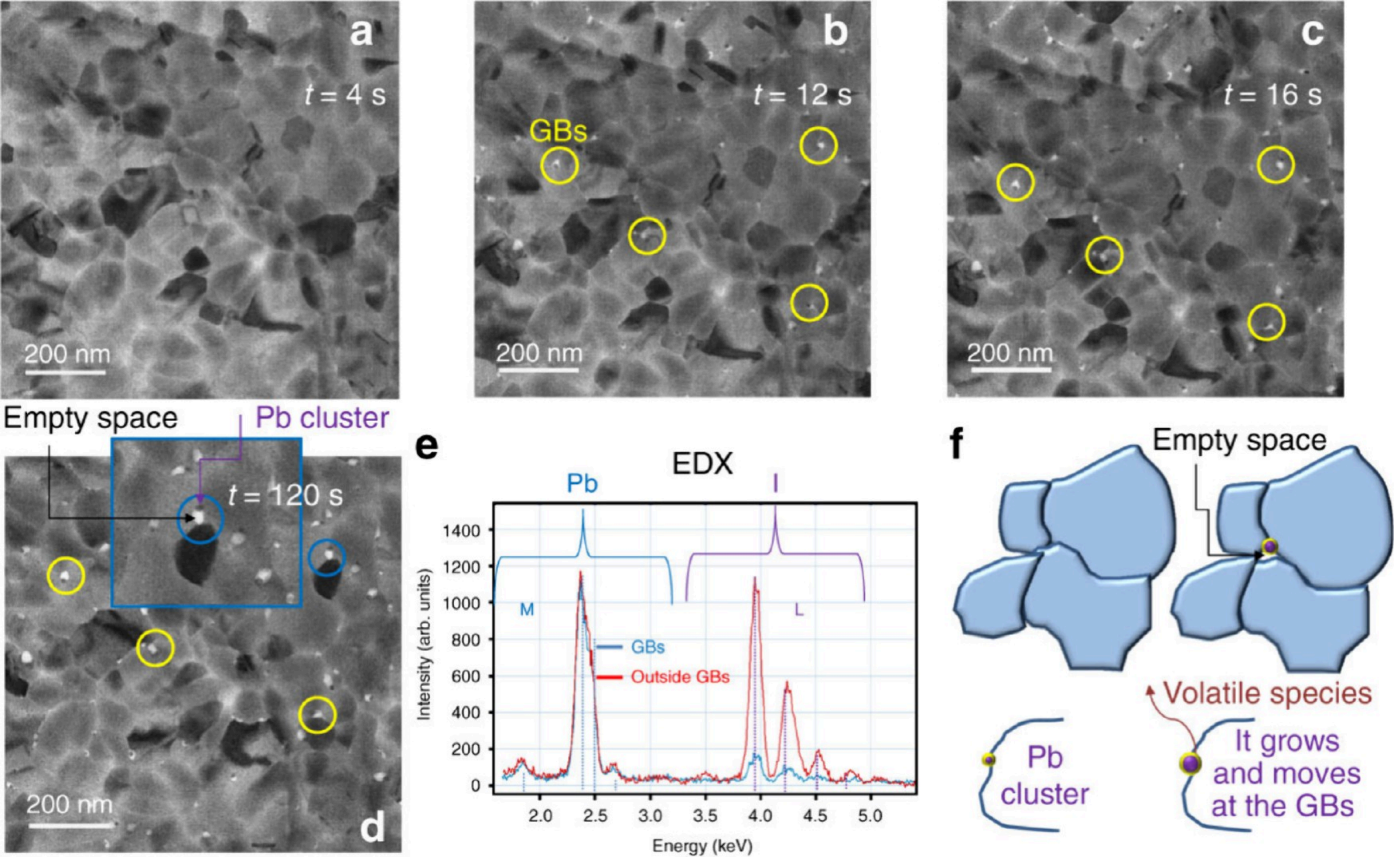
(a–d) Sequence of plane-view TEM images acquired over time at a fixed sample position. Each image was recorded with an electron dose rate of 1 e^–^ Å^–2^ s^–1^ and an acquisition time of 4 s. Yellow circles correspond to Pb clusters at the GBs. The inset in (d) shows a magnified region highlighting a cluster moved, leaving behind a void (linked white area). (e) EDX profiles, normalized to the most intense Pb peak. The vertical bars represent the expected relative intensities by Pb and I atoms. (f) Schematic illustration of Pb nanoclusters formation at the perovskite grain boundary. Reproduced from ref 48. CC BY 4.0.

Exposure to electron beams during microscopy induces localized heating and generates free electrons that interact with the perovskite lattice. These interactions can reduce Pb^2+^ to Pb^0^, especially in regions with existing halide vacancies or undercoordinated Pb^2+^. Notably, electron-beam-induced degradation does not uniformly affect the material, often leading to spatially confined defect sites. This highlights a crucial concern for nanoscale characterization techniques, where beam damage might alter material properties before meaningful data are acquired. Future work should investigate low-dose imaging methods or precoating strategies with beam-stable protective layers to mitigate these effects.

### Other Effects

#### Ion Beam

Perovskite–ion beam interactions, utilizing Ga^+^ or high-energy I^7+^ and Br^5+^ ions, lead to the formation of Pb^0^ in CsPbBr_3_ and MAPbBr_3_ perovskites due to the displacement of atoms from their lattice positions.^50,51^ The XPS measurements revealed an increase in Pb^0^ content as the irradiation conditions became more severe. On the other hand, argon gas cluster ion beam (GCIB) was shown to minimize Pb^0^ formation in MAPbI_3_ perovskite compared to conventional Ar^+^ sputtering. However, even soft cluster ion beams can induce Pb^0^ at the surface if exposure is prolonged or energy exceeds a threshold.^52^

#### H_2_O Effects

MAPbI_3_ perovskite decomposes to PbI_2_, Pb^0^, HI, and NH_3_ upon water vapor exposure.^53^ The MAPbI_3_ films showed Pb^0^ signal in the XPS measurements even before water exposure, which increased after the water exposure, and was accompanied by iodine loss. The initial Pb^0^ is mainly due to the decomposition of the lead precursor during the deposition and the increase in the Pb^0^ content after water exposure is due to water-induced degradation. Generally, moisture accelerates the degradation process by facilitating hydrolysis of the perovskite structure. Water initiates the decomposition of MAPbX_3_ to PbX_2_, followed by further reduction to Pb^0^ under external stressors.^26,45,54^

#### Applied Voltage

Biasing-induced changes in surface states and chemical composition may also contribute to the formation of Pb^0^.^55^ Specifically, applying bias voltages of 2 and 10 V for 10 min led to the appearance of a Pb^0^ chemical state on the surface of the MAPbBr_3_ SCs. The mechanism behind the formation of Pb^0^ under biasing conditions involves the breakage of the CH_3_NH_2_-molecular-defect-incorporated perovskite and the depletion of CH_3_NH_2_, MABr, and Br_2_ from the surface. As a result, Pb^0^ remains only on the surface after biasing. Recently, it was found that Pb^0^ formation under reverse bias is a critical degradation pathway, linked to electron injection and device electrochemistry.^56^

The formation of Pb^0^ in perovskite materials under various conditions highlights the ease of these materials to degrade and thus indicates the need for innovative strategies to enhance their stability. The degradation processes observed, such as those induced by ion bombardment, water vapor, and electrical biasing, point to the complexity of maintaining structural and compositional stability of perovskite films. To overcome this, one promising avenue is the development of perovskite materials by tailoring their chemical composition by incorporating more stable organic cations to reduce the formation of volatile species and hence can survive under operational conditions. Additionally, the use of passivation layers could help protect the perovskite materials from external factors that contribute to Pb^0^ formation.

## Influence of Synthetic Factors on Pb^0^ Formation

### Excess PbX_2_

Excess PbX_2_ in the perovskite films can be from intentionally added excess Pb precursor in the perovskite precursor solution to enhance device performance by improving charge extraction, or can be residual from the degradation of the halide perovskite films.^57^ Both Pb sources will result in the formation of Pb^0^ through the PbX_2_ photolysis. For example, the synthesis of halide perovskite films from different MAI and PbCl_2_ ratios showed that only at a high PbCl_2_ content (MAI:PbCl_2_ 1:1) that a signal in XPS measurements corresponding to Pb^0^ was detected, highlighting the importance of avoiding excess Pb precursor.^58^ Another work pointed out the dramatic effect of excess PbI_2_ by intentionally adding a 5 mol % extra PbI_2_ into the perovskite solution.^59^ The formation of Pb^0^ in metal halide perovskites (MAPbI_3_ or FA_1–*x*_MA_*x*_PbI_3_) was suggested to originate from the decomposition of the excess PbI_2_ rather than the perovskite under X-ray irradiation (Figure 7a). This was based on the observation of Pb^0^ impurity only in perovskite films with excess PbI_2_. The results indicate that MAPbI_3_ perovskite itself possesses good stability under long-term X-ray radiation, as compared with the one with excess PbI_2_. The formation mechanism of Pb^0^ involves the decomposition of PbI_2_ triggered by photon excitations with energies higher than around 2.51 eV, such as those provided by X-ray sources used in XPS instruments. This decomposition process can be described by the equations below.

$${\mathrm{PbI}}_{2}\overset{>2.51\;\mathrm{eV}}{\to }{\mathrm{Pb}}^{0}+I_{2}$$

$${\mathrm{MAPbI}}_{3}\overset{6.17\;\mathrm{eV}}{\to }{\mathrm{PbI}}_{2}+\mathrm{MAI}$$

**Figure 7:**
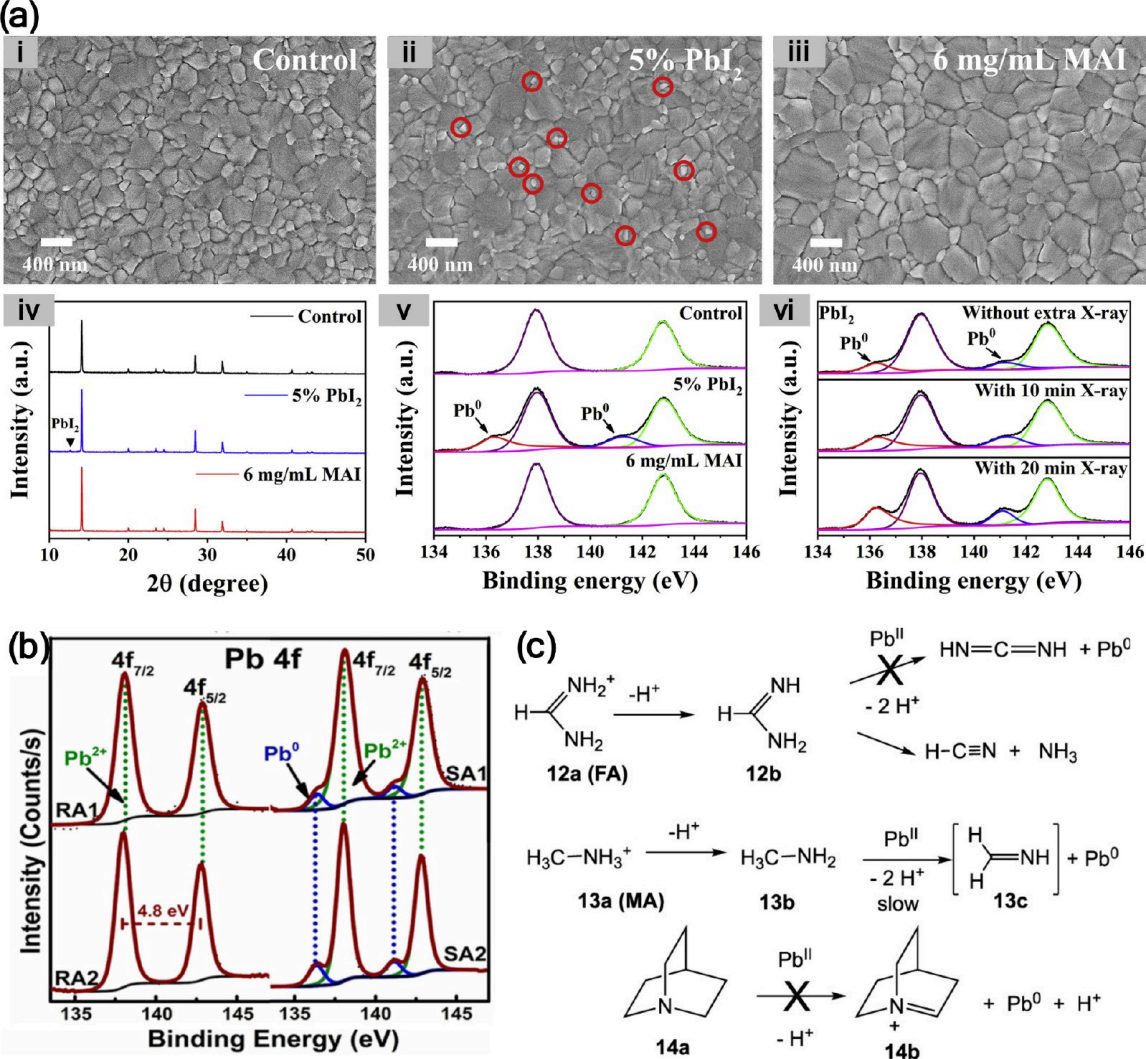
(a) (i–iii) Top-view SEM images of (i) a stoichiometric MAPbI_3_ perovskite film, (ii) MAPbI_3_ perovskite film with 5 mol % excess PbI_2_, (iii) MAPbI_3_ perovskite film with 5 mol % excess PbI_2_ and 6 mg/mL MAI post-treatment. (iv) Corresponding XRD patterns and (v) Pb 4f high-resolution XPS spectra of the perovskite films. (vi) Pb 4f high-resolution XPS spectra of PbI_2_ films with X-ray radiation for 0–20 min. Reproduced with permission from ref 59. Copyright 2022 Elsevier. (b) Pb 4f core-level spectra of perovskite films prepared using DMF/DMSO (RA1: fresh, RA2: aged) and using DMF (SA1: fresh, SA2: aged). Reproduced with permission from ref 62. Copyright 2018 Wiley-VCH. (c) Illustration of the unfavorable β-C–H elimination to form Pb^0^ for **12b**, where decomposition to HCN and NH_3_ is rapid; **13b**, where β-C–H elimination would be very slow because of the high energy of the parent imine (**13c**); and **14a**, where the resulting bridgehead olefin (**14b**) is highly strained. Reproduced from ref 61. Copyright 2021 American Chemical Society.

While small excesses of PbX_2_ can assist in defect passivation and improve crystallinity, significant excess leads to residual Pb-rich domains that are prone to reduction. The balance between beneficial and detrimental PbX_2_ content is delicate and highly system-specific. Process optimization should aim for minimal unreacted PbX_2_ while maintaining film uniformity and trap state suppression.

### Precursor Solution

In detailed mechanistic studies, it was proposed that the formation of Pb^0^ through the photolysis of the MAPbI_3_ perovskite does not provide a comprehensive set of degradation pathways.^60,61^ Instead, it was suggested that the origin of Pb^0^ formation in perovskite films is the chemical reactions induced by the soft Lewis acidity of Pb^2+^. Specifically, PbI_2_ reacts with methylamine additive, intentionally added to the precursor solutions, producing Pb-alkylamide bonds, which can be incorporated into the perovskite thin films.^60^ Subsequently, Pb^0^ formation occurs via a mechanism involving β-C–H proton transfer reactions of amido Pb species. The β-C–H bond mechanism for the formation of Pb^0^ in both PbI_2_ and in 2D perovskites (n-BA_2_Pb­(I/Br)_4_) involves several steps: (i) coordination of Pb^2+^ with an amine, forming a Pb^2+^–amine complex in the precursor solution; (ii) conversion of the Pb^2+^–amine complex to a Pb^2+^-amide complex through a proton-transfer reaction; and (iii) reduction of Pb^2+^ to Pb^0^ within the Pb^2+^–amide complex via β-hydrogen elimination, facilitated by the presence of a β-C–H bond in the amide ligand.^61^

Unlike several works reporting no perovskite degradation or Pb^0^ formation under dark conditions, it was demonstrated that PSCs stored for 90 days in dark under vacuum could still degrade and form Pb^0^.^62^ This behavior was highly dependent on the precursor solution; in particular, the solvent used in the fabrication of the MAPbI_3_ film. When a mixed solvent system of DMF/DMSO was used, neither fresh nor aged samples exhibited any Pb^0^ signal in the XPS measurements (Figure 7b). In contrast, when DMF was used as the sole solvent, Pb^0^ signal was detected in both fresh and aged samples, with an increased Pb^0^/Pb^2+^ ratio for the aged one. The degradation and formation of Pb^0^ highlights the critical role of solvent composition in governing the stability of perovskite films.

The precursor composition determines the nucleation and growth dynamics of the perovskite film. Solvents with strong coordination ability (e.g., DMSO, DMF) can stabilize Pb^2+^ temporarily but may also contribute to incomplete conversion or phase segregation if solvent is not fully removed. Future works should explore solvent systems with controlled volatility and minimal reactivity, as well as additive strategies to buffer against precursor instability.

### Perovskite Composition

The perovskite composition can significantly affect Pb^0^ formation. This could potentially be due to differences in the stability of the various perovskite lattices or the ability of some perovskite compositions to mitigate electron trapping and subsequent reduction of Pb^2+^ ions. For instance, starting with the A-site cation, perovskites containing FA and/or MA organic cations do not readily promote the reduction of Pb^2+^ to Pb^0^ (Figure 7c).^61^ In the case of MA, the reactive nature of the unsubstituted parent imine product (methylenimine) slows down the β-C–H elimination process, hindering the formation of Pb^0^. For FA compositions, FA autodecomposition to HCN and ammonia is rapid, which prevents the formation of Pb^0^.

Other studies on various perovskite compositions with different single A-site cation showed that FAPbI_3_ and all-inorganic perovskite compositions (CsPbI_3_ and CsPbBr_3_) exhibited superior radiation hardness under ultrahigh doses of γ rays and light exposure, respectively, with no Pb^0^ formation (Figure 8a).^8,27^ In contrast, under high flux density X-ray irradiation, CsPbBr_3_ showed signs of Pb^0^ formation.^63^ This was attributed to a direct degradation of the lead halide cage, which is also reflected in the Fermi edge in the valence band spectra (Figure 8b).

**Figure 8:**
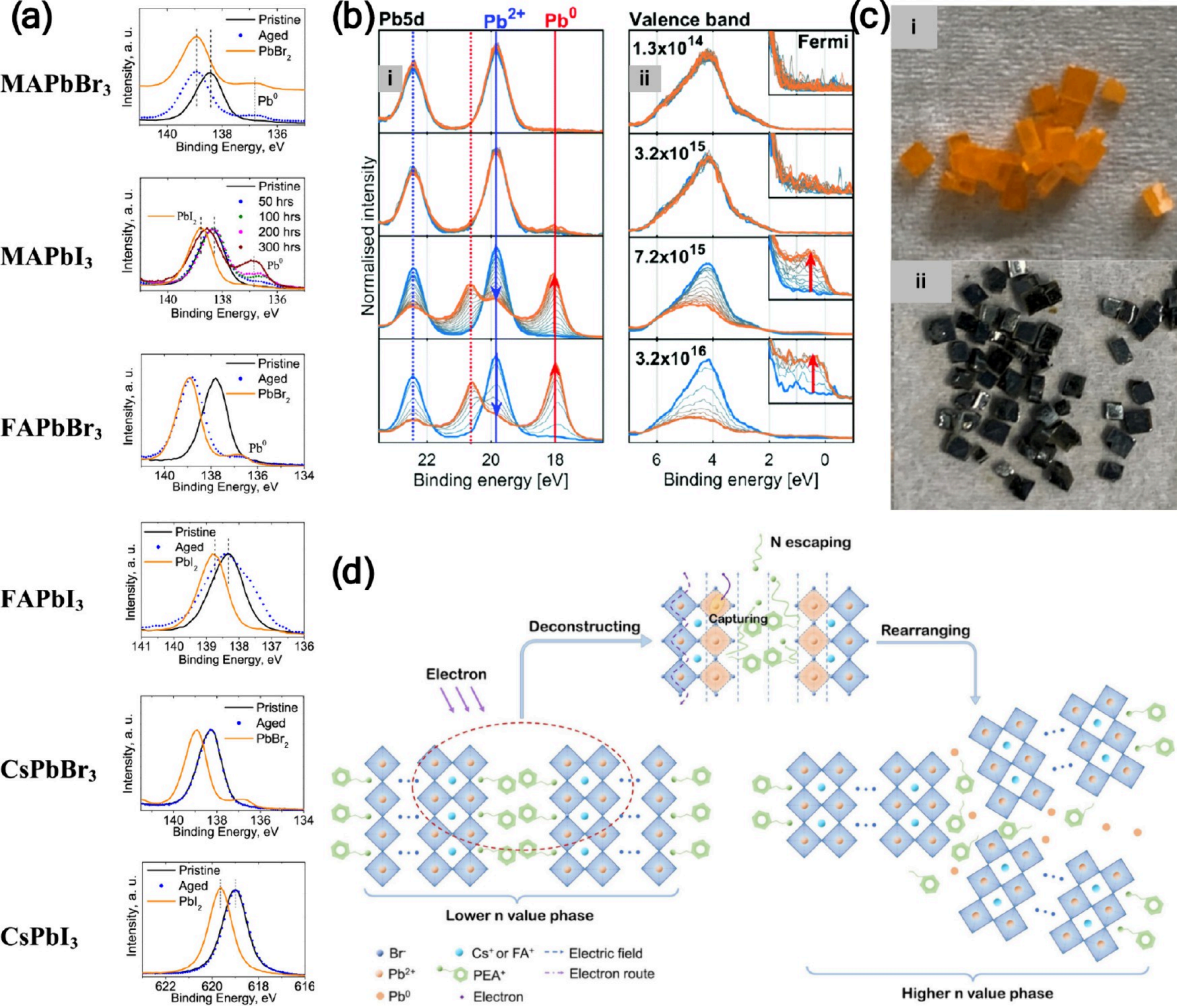
(a) XPS of Pb 4f spectra of various perovskite films after light soaking (∼100 mW/cm^2^, ∼70 °C) for 300 h. Reproduced from ref 8. Copyright 2019 American Chemical Society. (b) Pb 5d and valence band spectra recorded for CsPbBr_3_ perovskite as a function of time (blue: start, orange: end) under various X-ray flux densities. Reproduced from ref 63. CC BY 3.0. (c) Photographs of DMA/MAPbBr_3_ crystals grown using (i) low PbBr_2_ concentrations (0.4 M) and (ii) high PbBr_2_ concentrations (1.2 M). Reproduced from ref 12. Copyright 2023 American Chemical Society. (d) Schematic diagram illustrating the proposed degradation mechanism in PEA^+^-doped reduced-dimensional perovskites. Reproduced with permission from ref 64. Copyright 2023 Royal Society of Chemistry.

The use of mixed A-site cations (e.g., MA and DMA) facilitated the formation of Pb^0^ in mixed DMA/MAPbBr_3_ perovskite crystals (Figure 8c) when crystallized under ambient light conditions and high PbBr_2_ concentration.^12^ Interestingly, the presence of the mixed A-site cations was a must for forming higher Pb^0^ content, which resulted in the transforming of the DMA/MAPbBr_3_ crystals into black-colored crystals. On the other hand, using a single organic cation, MA or DMA, under similar crystallization conditions did not result in a high Pb^0^ content, and no blackening was observed. In the Cs_0.17_FA_0.83_PbI_3_ perovskite, the formation of Pb^0^ is probably due to the radiolysis of the organic cation, leading to the formation of organic degradation products and the collapse of the perovskite structure.^63^

The A-site cation was also found to be the initiator for the Pb^0^ formation in quasi-2D perovskites through the decomposition/escape of the large organic cation (PEA^+^) as illustrated in Figure 8d.^64^ The escape of positive charge centers (−NH_3_^+^) of the organic spacer molecules results in exposed surface that is prone to capturing electrons, facilitating the reduction of Pb^2+^ to Pb^0^. This was confirmed by testing 3D CsPbBr_3_ lacking any PEA^+^ cations, which showed no sign of Pb^0^ after electron beam irradiation.

This effect of the perovskite composition on Pb^0^ formation is further influenced by the choice of X-site halogen anion. Iodide-based perovskites are more basic than bromide-substituted species, leading to greater Pb^0^ formation (Figure 9a).^61^ Compositions with different A- and X-site ions APbX_3_ (A = MA, FA, or Cs and X = Br or I) were examined under white light illumination to investigate their photostability.^8^ The photostability of these perovskites are in the following order MAPbBr_3_ < MAPbI_3_ < FAPbI_3_ < FAPbBr_3_ < CsPbI_3_ < CsPbBr_3_. However, this is not directly related to the formation of Pb^0^. Instead, Pb^0^ formation depends on the initial decomposition product that are PbI_2_, which undergoes photolysis to Pb^0^, and PbBr_2_ which resists reduction to Pb^0^ due to bromide’s higher oxidation potential compared to iodide. This is in line with other work that confirmed no Pb^0^ was formed through direct decomposition of MAPbBr_3_ under illumination or in the dark under low heating conditions.^29^ Unlike MAPbI_3_, which forms several gaseous products upon decomposition, MAPbBr_3_ decomposes into CH_3_NH_2_ and HBr only:

$${\mathrm{MAPbBr}}_{3}\left(s\right)\overset{h\nu \;\mathrm{or}\;\Delta }{⇄}{\mathrm{PbBr}}_{2}\left(s\right)+{\mathrm{CH}}_{3}{\mathrm{NH}}_{2}\left(g\right)+\mathrm{HBr}\left(g\right)$$

This suggests MAPbBr_3_ is more stable than MAPbI_3_, which is in agreement with previous works.^65,66^

**Figure 9:**
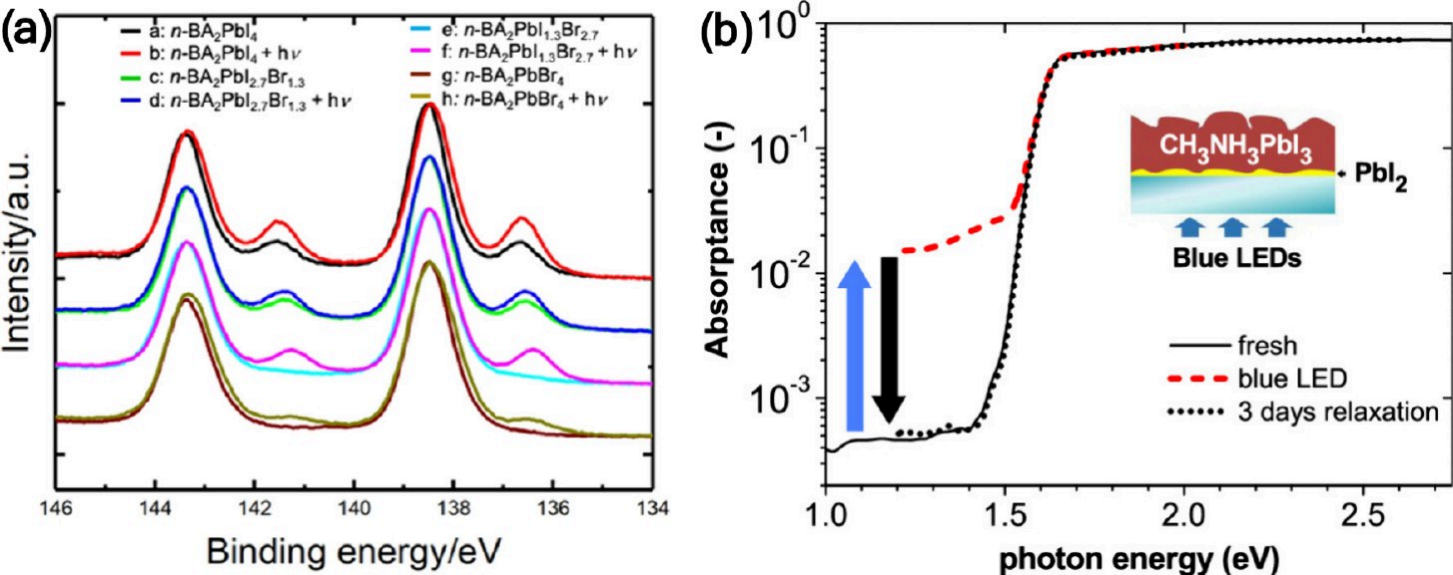
(a) XPS of Pb 4f spectra illustrating the effect of *n*-butylammonium (*n*-BA^+^) on the reduction of Pb^2+^ to Pb^0^ under dark conditions (18 h) and after light exposure (375 nm; ∼25 mW/cm^2^), for 18 h. Spectra are shown for *n*-BA_2_PbI_4_ in the dark, irradiated *n*-BA_2_PbI_4_, mixed halide species *n*-BA_2_PbI_2.7_Br_1.3_ in the dark, irradiated *n*-BA_2_PbI_2.7_Br_1.3_, *n*-BA_2_PbI_1.3_Br_2.7_ in the dark, irradiated *n*-BA_2_PbI_1.3_Br_2.7_, *n*-BA_2_PbBr_4_ in the dark, and irradiated *n*-BA_2_PbBr_4_. (b) Photothermal deflection spectroscopy (PDS) showing the spectral response of a perovskite sample before and after 30 min of blue LED irradiation. Reproduced with permission from ref 68. Copyright 2019 American Chemical Society.

The formation of Pb^0^ in mixed-halide perovskites originates from the aggregation of vacancies, as predicted by Monte Carlo simulations.^67^ These vacancies tend to migrate and accumulate, promoting the reduction of Pb^2+^ to Pb^0^, while hole carriers oxidize iodide ions. The presence of bromide-containing mixed-halide perovskites exacerbates this degradation compared to Br-free compositions. This is because of different reasons, including: (i) bromide incorporation introduces additional defects into the perovskite lattice, which can serve as nucleation sites for the aggregation of vacancies, accelerating the formation of Pb^0^ clusters; and (ii) bromide-containing perovskites may exhibit higher defect mobility compared to bromide-free compositions, which facilitates the migration and aggregation of defects.

The selection of A, and X-site ions also plays a role in determining a perovskite’s resistance to Pb^0^ formation. In particular, all-inorganic systems like CsPbX_3_ show greater thermal and photochemical stability than MA- or FA-based perovskites, due to reduced volatility. Additionally, replacing I^–^ with Br^–^ improves resistance to light-induced halide loss and phase segregation. Exploring mixed-metal (e.g., Bi–Pb) and mixed-halide systems offers opportunities to reduce instability, though care must be taken to avoid introducing new redox-active centers.

### Fabrication Technique

In a two-step spin-coating method of perovskite films, residual PbI_2_ will often be present, which is considered the origin of Pb^0^.^68^ In particular, when PbI_2_ is subjected to blue LED illumination, Pb^0^ is formed and sub-bandgap absorption increases (Figure 9b). Hence, it is recommended to utilize the single-step spin-coating method for the fabrication of MAPbI_3_ thin films. Additionally, preventing the illumination of PbI_2_ during the fabrication process can also help eliminate the formation of Pb^0^. In another study, different variations of the two-step spin-coating fabrication technique were explored to investigate the evolution of residual PbI_2_ content in perovskite films and its impact on the stability and performance of perovskite solar cells.^6^ Among these variations, the spin coating of FAI-MAI on unannealed PbI_2_ films exhibited perovskites with reduced Pb^0^ content, as these conditions resulted in a modest quantity of unreacted PbI_2_. Despite using a one-step spin coating method for the fabrication of triple cation (CsFAMA) mixed halide (BrI) perovskite, Pb^0^ was detected in the perovskite films and was attributed to the photolysis of unreacted PbI_2_ under continuous light illumination.^69^ Adding to the complexity, it was reported that the formation of Pb^0^ depends on the degree of coverage of the MAPbI_3–*x*_Cl_*x*_ films on top of the TiO_2_/FTO/glass substrates.^70^ While fully covered areas showed no sign of Pb^0^ in the XPS measurements, a partially covered area showed a high Pb^0^ signal reaching 2:1 Pb^2+^:Pb^0^. Therefore, it was suggested that, regardless of the reason behind the formation of Pb^0^, this process happens only or at least is initiated locally. Extensive antisolvent toluene treatment of MAPbI_3_ films results in iodine loss, and hence the formation of Pb^0^.^71^ While the iodine removal by toluene was found to be 10 times more under illumination, this study challenges the assumption that Pb^0^ originates only during postdeposition degradation (e.g., light, moisture, or heat exposure). It reveals that the fabrication process itself, particularly the antisolvent choice, can be a source of intrinsic metallic lead.

The method of film deposition, whether spin-coating, blade coating, or vapor deposition, affects crystallization kinetics, film morphology, and ultimately defect density. Rapid crystallization can trap solvent or result in poor grain connectivity, both of which promote Pb^0^ formation. On the other hand, slow nucleation may enable solvent coordination release (e.g., hot-casting or vacuum-assisted methods) that is crucial for high-quality films with reduced defect pathways.

### Metal Contacts

The impact of different metal contacts on the formation of Pb^0^ in PSCs was also explored.^72,73^ The Pb^0^ formation was recorded with several metal contacts (Al, Ti, Cr, Ag, and Au) with lower Pb^0^ content for Ag and Au compared to the other metal contacts.^72^ The formation of Pb^0^ occurs at the metal/MAPbI_3_ interface originating from redox reactions between the deposited metal layers and iodine ions from the MAPbI_3_ surface.^73^ This formation mechanism involves the reduction of Pb^2+^ in MAPbI_3_ to Pb^0^ due to charge transfer between the metal contact and the halide perovskite. Furthermore, the thermodynamically favorable reactions between the metal and iodine result in the formation of metal–iodine compounds and the generation of Pb^0^ at the interface.

Direct contact between perovskites and reactive metal electrodes (e.g., Ag, Al) can induce redox reactions, especially under bias or illumination. Such reactions release I_2_, leading to Pb^2+^ reduction and the formation of Pb^0^ clusters near the interface. The introduction of diffusion barriers, buffer layers, or stable interfacial materials such as MoO_3_, TiO_2_, or SnO_2_ helps mitigate metal ion migration and preserve the redox balance at contacts.

In summary, it has become clear that intrinsic factors, such as the precursor solution, play a critical role in the perovskite degradation pathway and Pb^0^ formation. Therefore, optimizing the precursor composition, including precise control of PbI_2_ content, and refining the fabrication processes such as the spin-coating techniques, are essential to suppress the formation of Pb^0^. Understanding the correlation between the presence of excess PbI_2_ and the different fabrication conditions (e.g., annealing temperatures and spin-coating methods) will assist in the development of more robust perovskite materials. The discovery that Pb^0^ can form in halide perovskites not only by light or X-ray exposure but also through complex chemical reactions involving Pb^2+^ and organic components such as aliphatic amines adds a new layer of complexity to the challenge of overcoming the instability issue of halide perovskites. The β-C–H proton transfer mechanism highlights the importance of understanding the chemical environment in the perovskite precursor solution and how the choice of organic cations, solvents, and fabrication conditions can influence the Pb^0^ formation. The role of the perovskite composition in determining its vulnerability to Pb^0^ formation is evident from the increased stability of Cs-based perovskites compared to MA- or FA-based counterparts. The differences observed in the behavior of iodide- versus bromide-based perovskites further emphasize the need for a detailed understanding of halide chemistry and its impact on degradation pathways. Furthermore, the study of metal-perovskite interfaces reveals that identifying metals that minimize redox reactions with iodine ions or developing protective interlayers could significantly reduce the formation of Pb^0^. This suggests that future work should focus on the careful design of perovskite compositions and fabrication methods involving chemicals that could enhance the perovskite stability and suppress the Pb^0^ formation.

## Reversibility

It is observed that the decomposition of the perovskites could be reversible, particularly as Pb^0^ along with I_2_ can reform PbI_2_.^29^ However, some decomposition products (NH_3_ and CH_3_I) would be described as irreversible. Hence, it is recommended to identify pathways that could prevent the formation of such decomposition products to allow the regeneration of MAPbI_3_ during the off-illumination time frame.

In the work investigating the degradation of (FAPbI_3_)_0.85_(MAPbBr_3_)_0.15_ using a 515 nm laser, it was observed that when the laser is switched off, the amount of Pb^0^ initially decreases in the first few minutes and then stabilizes at a constant amount, regardless of the laser power.^39^ The final concentration of Pb^0^ after turning off the laser is approximately 44% of the concentration measured immediately before the laser is turned off. The reduction in Pb^0^ content could be attributed to the migration of Pb^0^ within the film. Further, within the film, any generated I_2_ could potentially be retained, fostering the reformation of the perovskite in the absence of light. Similarly, experiments were the degraded perovskite film containing Pb^0^ was left undisturbed in vacuum showed that, over time, the Pb^0^ gradually reoxidized back to Pb^2+^, indicating a reversible transformation.^40^ The reversibility of the reaction was also validated by the absorptance of the films, which returns to preillumination levels after keeping the sample in the dark for a certain period (Figure 9b).^68^

A postannealing procedure was utilized to reduce the Pb^0^ content in MAPbI_3_ thin films, confirming that the formation of Pb^0^ is a reversible process (Figure 10a).^74^ The optimum conditions for this process is postannealing for 1 h at 70 °C to avoid degradation of MAPbI_3_. The decrease in Pb^0^ content is suggested to be through its incorporation in the perovskite structure. In the case of MAPbBr_3_ SCs, the exposure to Br_2_ vapor showed that the Br-vacancies can be passivated, which can effectively reverse the Pb^0^ formation process in the SCs.^75^ Additionally, the introduction of Br_2_ vapor was found to enhance the p-doping of the crystals, further indicating the removal of Pb^0^.

**Figure 10:**
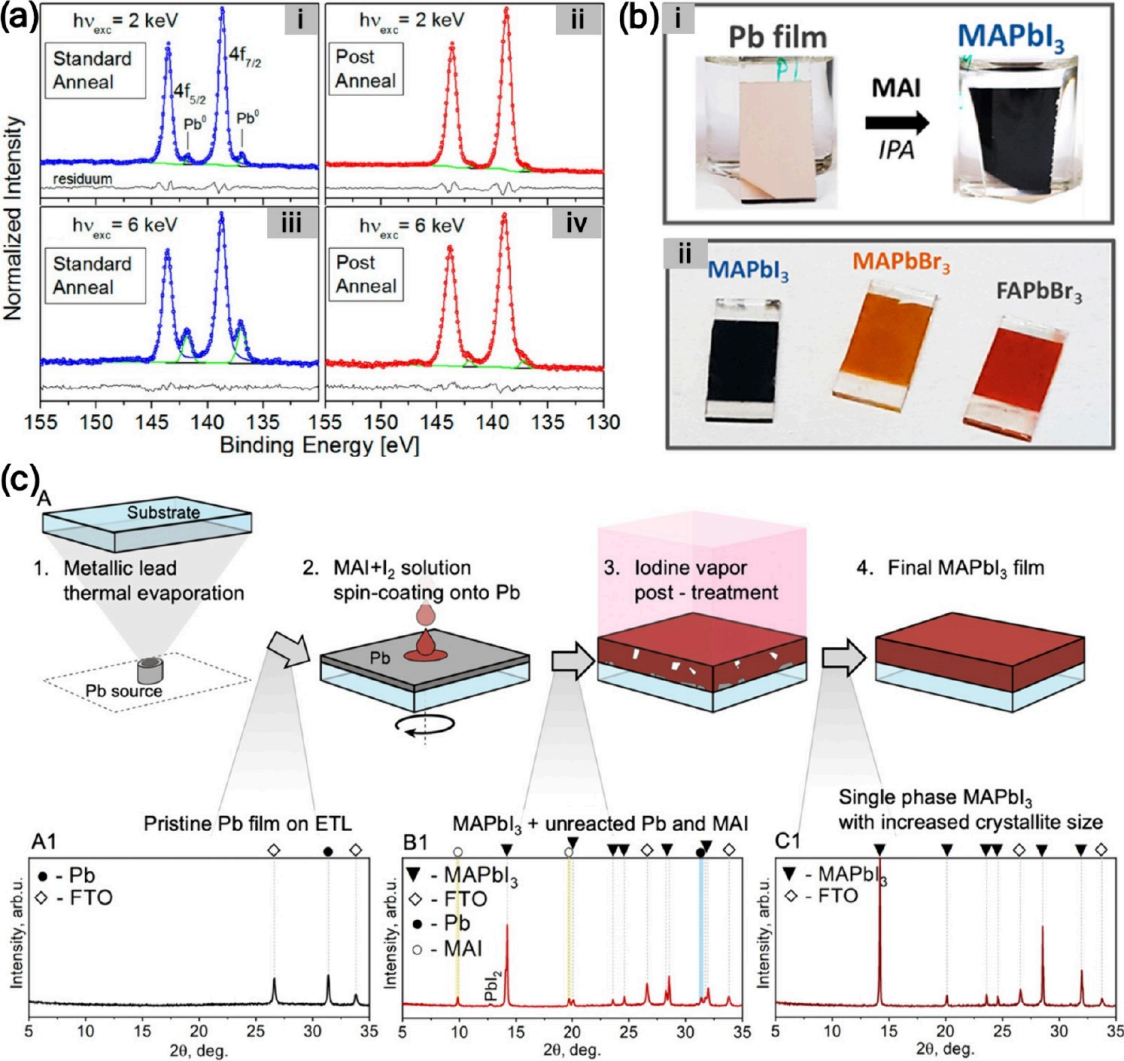
(a) High-resolution Pb 4f_5/2_ and 4f_7/2_ XPS spectra (dots) acquired using excitation photon energies of (i, ii) 2 and (iii, iv) 6 keV for a 60 nm thick MAPbI_3–*x*_Cl_*x*_ perovskite film on TiO_2_ subjected to two different annealing treatments: (i, iii) standard annealed in N_2_ (90 °C for 150 min) and (ii, iv) postannealed in air (70 °C for 14 h). Reproduced from ref 74. Copyright 2015 American Chemical Society. (b) Photographs of (i) Pb film on d-TiO_2_/FTO/glass substrate before and after treatment with MAI dissolved in IPA and (ii) halide perovskite films after treatment of similar Pb films with solutions of (from left to right) 50 mM MAI (∼2 h@ 20 °C), 70 mM MABr (4 h @ 50 °C), and 70 mM FABr (5 h @ 50 °C). Reproduced from ref 78. Copyright 2017 American Chemical Society. (c) Schematic illustration of perovskite synthesis from metallic Pb films via oxidation by reactive polyhalide solution and iodine vapor. (A1–C1) XRD patterns of the pristine Pb@FTO film, the MAPbI_3_ and precursor mixture film after spin-coating the MAI + I_2_ mixture solution, and the final MAPbI_3_ film after 9 min of saturated I_2_ vapor treatment, respectively. Reproduced from ref 79. Copyright 2020 American Chemical Society.

A significant advancement demonstrated the successful synthesis of MAPbI_3_ perovskite through fabricating a thin film of Pb^0^ followed by spin-coating of a MAI layer.^76^ Similarly, Pb^0^ was used as a precursor for the formation of single and mixed A-site cation perovskites; a nanoscale layer of Pb^0^ was coated with MAI or CsI/MAI/FAI and then was exposed to iodine vapor.^70^ Additionally, Pb^0^ was also converted to pure or mixed cation/anion perovskite films by reacting it with polyiodide melts,^77^ with alcoholic solutions of Cs, MA, and FA (Figure 10b),^78^ or solutions of MA and FA polyhalides dissolved in isopropanol (Figure 10c).^79^

The discovery of the reversibility of Pb^0^ formation in halide perovskites could pave the way for improving the perovskite stability. The ability to convert Pb^0^ back into the active perovskite phase offers a potential route for long-term material stability under operational conditions. In future research, the focus should be directed toward understanding the mechanism of this reversible transformation in greater detail, aiming to identify the optimal conditions under which Pb^0^ can be reoxidized to Pb^2+^ and reincorporated into the perovskite structure. Simultaneously, researchers should explore the development of materials and fabrication techniques that can inhibit the formation of the irreversible degradation products, such as NH_3_ and CH_3_I.

## Suppression of Pb^0^

Suppressing the formation of Pb^0^ in perovskite compositions is crucial for enhancing the stability and performance of perovskite-based devices. Various strategies have been explored to achieve this, including using mixed cations and halides, incorporating bulky organic cations, and using additives in the precursor solution and interlayers in the device.

### Perovskite Composition

One of the recommended strategies involves the manipulation of the A-site cations. The replacement of the MA cation with mixed FA/Cs cations or the use of triple CsFAMA cations in the iodide-based films helps avoiding the formation of the irreversible decomposition products (NH_3_ and CH_3_I)^29^ and provide the best structural stability and hence possessed the lowest Pb^0^ content (Figure 11a).^33^ Similarly, mixed A-site cations involving Cs in Br-based and mixed bromide-iodide perovskites was also successful in eliminating Pb^0^.^67,80,81^ The Pb^0^ formation in these perovskites was suppressed primarily through the addition of Cs^+^ ions in the perovskite composition (Figure 11b). It was found that the Cs^+^ ions enhanced crystallinity, decreased defect density, and limited ion migration, hence stabilizing the perovskite structure and reducing the Pb^0^ formation. In addition to structural benefits, these mixed-cation compositions also exhibit higher resistance to halide migration and phase segregation, which are key kinetic factors that often trigger degradation. In contrast, single-cation systems such as MAPbI_3_ or FAPbI_3_ are more susceptible to decomposition due to their lower lattice stability and volatile degradation products. Additionally, the optimization of halide ratio in the perovskite composition, particularly by increasing the proportion of iodide relative to bromide, minimized the formation of Pb^0^. Maintaining a balanced halide composition helps prevent phase segregation and instability, contributing to the prevention of Pb^0^ formation.

**Figure 11:**
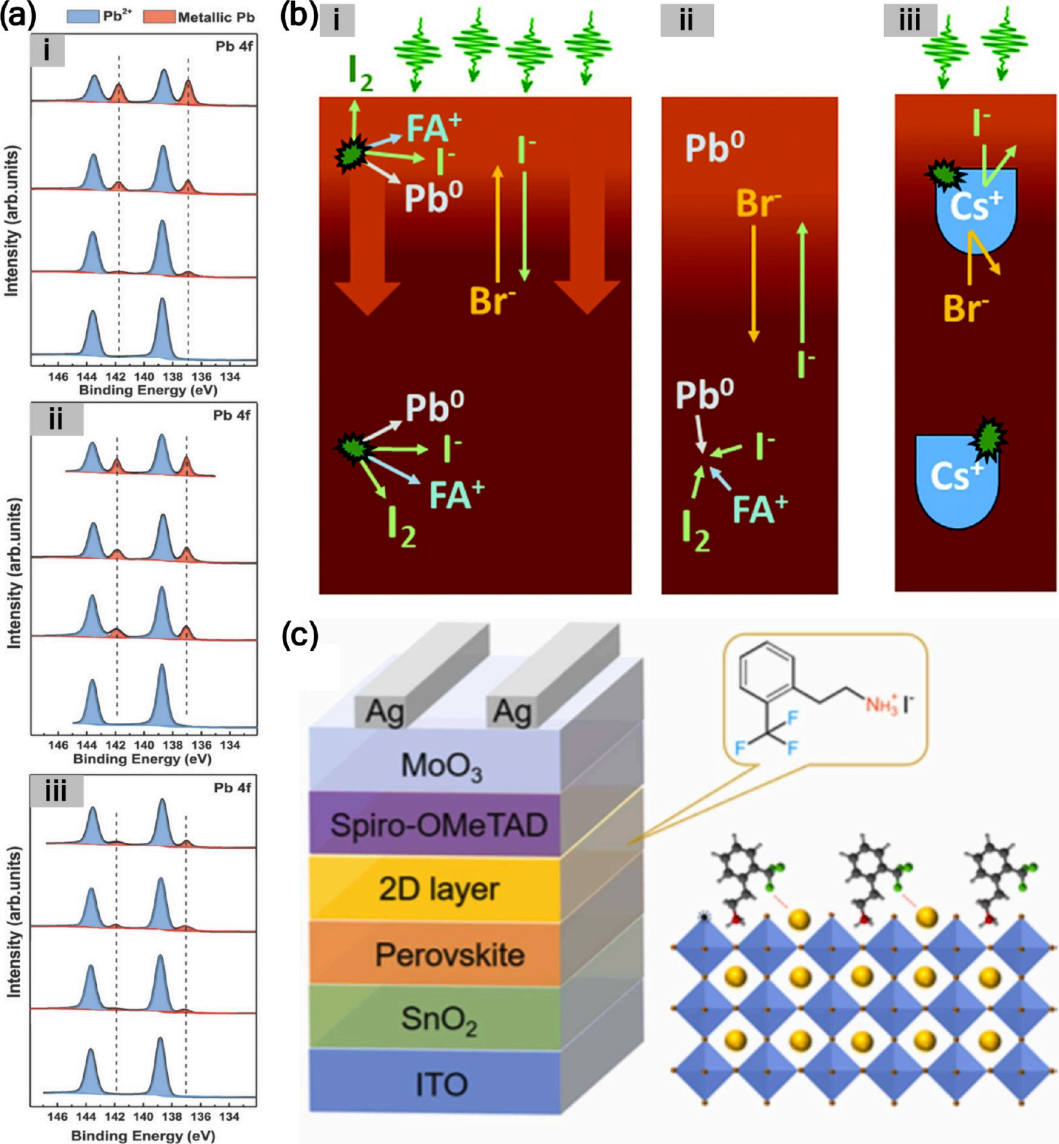
(a) Evolution of XPS core-level spectra of the (i) MA, (ii) FAMA, and (iii) CsFAMA perovskite films as a function of in situ illumination time. Reproduced with permission from ref 33. Copyright 2018 Wiley-VCH. (b) Schematic representation of the light-induced degradation and ion migration and recovery process: (i) formation of Pb^0^ with some I_2_ products escaping to vacuum and migration of halides and organic cations to the surface layer; (ii) recovery processes postillumination: reintegration of Pb^0^, and halides exchange between surface layer and perovskite; (iii) the protective role of Cs^+^ at optimum Br:I ratio: suppressing the formation of Pb^0^ and I_2_ and blocking halide and organic cation migration. Reproduced from ref 81. CC BY-NC 3.0. (c) Schematic of the device structure and the perovskite film composition. Reproduced with permission from ref 82. Copyright 2022 Elsevier.

Employing bulky organic cations with greater thermal stability, such as phenyltrimethylammonium iodide (PTMAI) can reduce the perovskite thermal decomposition, hence suppressing the formation of Pb^0^.^47^ 2-trifluoromethylphenylethylamine hydroiodide (2-CF_3_-PEAI) was utilized to react with Pb^0^ forming a thin layer of 2D perovskite ((2-CF_3_-PEA)_2_PbI_4_) atop the 3D perovskite structure, hence eliminating Pb^0^ without impeding charge carrier transport (Figure 11c).^82^

In conclusion, the synthesis of mixed cation perovskites, such as the triple cation CsFAMA, represents a promising route toward reducing the formation of Pb^0^ and thus improving the stability and performance of perovskite-based devices. Similarly, the ratio of iodide to bromide is another crucial factor in controlling Pb^0^ formation. By optimizing this ratio, researchers can mitigate phase segregation and instability, which are common causes of perovskite degradation. In addition to these compositional engineering strategies, the use of bulky organic cations like PTMAI and 2-CF_3_-PEAI represents a novel approach to suppressing Pb^0^ formation. These organic cations form a thin 2D perovskite layer on top of the 3D perovskite, adding an additional protective layer that enhances the stability of the perovskite material without hindering its electronic properties. Future research should focus on exploring a broader range of cation combinations and halide ratios to identify the most effective compositions for suppressing Pb^0^ formation. Additionally, advancements in understanding the interactions between these cations and the perovskite lattice at the molecular level could lead to the design of materials with tailored properties that resist degradation more effectively.

### Perovskite Precursor Solution

One strategy to suppress the Pb^0^ content, without introducing foreign cations/halides, is through varying the precursor ratio itself.^83^ For example, at a 1:1 MABr:PbBr_2_ ratio, bromine loss or incomplete reaction between the precursors leads to the formation of Pb^0^, which results in lower PL due to the increased nonradiative decay. On the other hand, MAPbBr_3_ films spin-coated from a 1.05:1 precursor ratio showed no sign of Pb^0^, increased PL intensity, and longer average PL lifetime (Figure 12a), confirming enhanced radiative recombination with respect to the 1:1 films. Interestingly, despite the absence of a Pb^0^ signal in the XPS measurements for the 1.05:1 films, the films remained Br-deficient with a Br:Pb ratio of 2.64:1. However, this was still higher than the 2.32:1 ratio reported for the 1:1 films. The use of excess MA precursor with respect to the PbX_2_ to suppress the formation of Pb^0^ was also utilized for MAPbI_3_ films.^84^ Similarly, adding excess CsBr in the fabrication of CsPbI_2_Br helped suppress the formation of Pb^0^ and mitigated halide phase segregation in the mixed halide perovskite.^41^ The excess CsBr accumulated at the grain boundaries and quenched the formation of PbI_2_ during Pb^0^ and I_3_^–^ recombination, thereby promoting the regeneration of the mixed halide perovskite CsPbI_2_Br. Additionally, CsBr can undergo ion exchange reactions with photochemically generated CsPbI_3_, facilitating the reformation of the mixed halide perovskite and reducing the accumulation of Pb^0^ and I_3_^–^ defects at grain boundaries.

**Figure 12:**
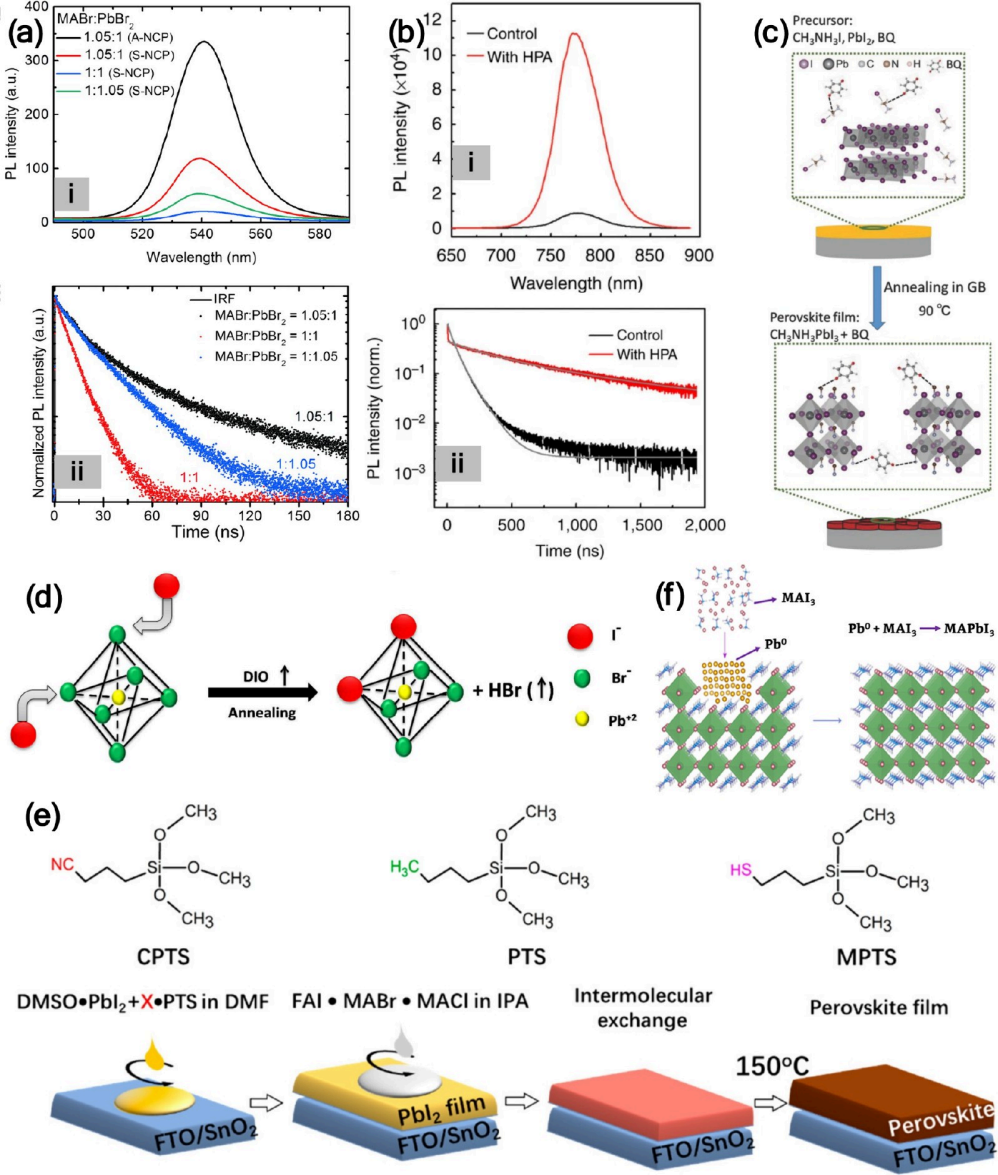
(a) (i) Steady-state PL spectra of MAPbBr_3_ nanograin layers with varying molar ratio of MABr:PbBr_2_ and (ii) corresponding PL lifetime curves. Reproduced with permission from ref 83. Copyright 2015 American Association for the Advancement of Science. (b) (i) Steady-state and (ii) TRPL spectra for perovskite thin films deposited on glass prepared from precursor solutions with and without (control) HPA. Reproduced from ref 3. CC BY 4.0. (c) Schematic of the proposed perovskite film formation process. Reproduced with permission from ref 88. Copyright 2016 Wiley-VCH. (d) Diagram illustrating the ion exchange process by the addition of DIO into the perovskite precursor solution. Reproduced with permission from ref 91. Copyright 2018 Royal Society of Chemistry. (e) Chemical structures of CPTS, PTS, and MPTS additives along with the schematic illustration of the formation process of perovskite films based on a sequential deposition method; X indicates cross-linking terminal groups (−CN, −CH_3_, and −SH) of CPTS, PTS, and MPTS, respectively. Reproduced from ref 92. Copyright 2019 American Chemical Society. (f) The use of MAI_3_ as an additive to suppress the formation of Pb^0^. Reproduced from ref 7. Copyright 2021 American Chemical Society.

Changing the Pb precursor to Pb­(NO_3_)_2_ allows the use of aqueous solution for a more environmentally friendly route. The use of halide-free nanofluids (NFs), in particular PbCO_3_, in this aqueous solution suppresses the formation of Pb^0^ by regulating the crystallization of the perovskite material through accelerating the transformation of PbI_2_ into the perovskite.^85^ The reaction takes place as shown below:

$${\mathrm{PbCO}}_{3}+2\mathrm{MAI}\overset{\Delta }{\to }{\mathrm{PbI}}_{2}+{\mathrm{MA}}_{2}{\mathrm{CO}}_{3}$$

$${\mathrm{PbI}}_{2}+\mathrm{MAI}+{\mathrm{MA}}_{2}{\mathrm{CO}}_{3}\overset{\Delta }{\to }{\mathrm{MAPbI}}_{3}+{\mathrm{CO}}_{2}↑+H_{2}O↑+2{\mathrm{CH}}_{3}{\mathrm{NH}}_{2}↑$$

net equation:

$${\mathrm{PbCO}}_{3}+3\mathrm{MAI}\overset{\Delta }{\to }{\mathrm{MAPbI}}_{3}+{\mathrm{CO}}_{2}↑+H_{2}O↑+2{\mathrm{CH}}_{3}{\mathrm{NH}}_{2}↑$$

Following a study by Guo et al.^86^ exploring the use of terpineol in the fabrication of MAPbI_3_ films with enhanced moisture stability, the substitutional growth of MAPbI_3_ in polar protic alcohols was investigated.^9^ The use of alcohols as solvents resulted in Pb^0^-free MAPbI_3_ and was attributed to the protic nature of the alcohols, which prevented the oxidation of I^–^ to I_2_. Therefore, the halide vacancies are reduced, stoichiometric MAPbI_3_ is formed, and Pb^0^ is eliminated.

Unlike strategies that introduce foreign cations or halides, changing the perovskite composition and properties, fine-tuning the precursor ratio can directly address the formation of Pb^0^. This approach can lead to more efficient material synthesis without complicating the perovskite composition with additional elements. In addition to precursor ratio manipulation, alternative strategies such as incorporating halide-free nanofluids (e.g., PbCO_3_) and using polar protic alcohols as solvents are also effective in suppressing Pb^0^ formation. These methods not only suppress Pb^0^ formation but also provide a route for enhanced perovskite properties, such as moisture stability and improved crystallization. The exploration of these methods in various perovskite compositions and under different environmental conditions will likely yield valuable insights into achieving long-term stability in perovskite devices.

### Additives

#### Organic Additives in the Precursor Solution

The first report on using an additive in the precursor solution to reduce the Pb^0^ content was published in 2015.^3^ Using hypophosphorous acid as an additive decreased the Pb^0^ content from 10.9% to 5.6%. This decrease is due to HPA reducing I_2_ back to I^–^, hence forming a more balanced Pb:I stoichiometry. The reduction in Pb^0^ content and halide vacancy was reflected in the more intense PL, higher PLQY (11.6% vs 0.84%) and reduced trap states (1.58 × 10^16^ to 3.25 × 10^15^) as per the PL lifetime measurements (Figure 12b).

Several other organic additives were also reported to successfully reduce the Pb^0^ content in the perovskite materials. Benzoquinone (BQ), a weak oxidant that was previously used in the synthesis of PbS QDs to suppress the formation of Pb^0^,^87^ was successfully utilized as an additive in the MAPbI_3_ precursor solution (Figure 12c).^88^ A reduction in the Pb^0^ from 8.9% to 3.2% in the fabricated MAPbI_3_ films was recorded as indicated by XPS measurements. 2,3,5,6-Tetrafluoro-7,7,8,8-tetracyanoquinodimethane (F4TCNQ), a strong electron acceptor, was also used to reduce the Pb^0^ content in MAPbI_3_ films based on the high interaction between F4TCNQ and undercoordinated Pb atoms in the perovskite.^89^ F4TCNQ as an additive resulted in a lower dark current, which is beneficial for photodetectors.^90^ The suppression of Pb^0^ in MAPbBr_3_ films was achieved by using 1,8-diiodooctane (DIO) as an additive. Although the suppression mechanism was not fully explained, it was demonstrated that halide exchange between bromine in the perovskite and iodine in the DIO takes place (Figure 12d).^91^ Efficient PSCs were fabricated through utilizing (3-mercaptopropyl)­trimethoxysilane (MPTS) as an additive in the precursor solution (Figure 12e).^92^ Among the advantages that MPTS provided was its ability to passivate defects in the perovskite film, in particular, the iodine vacancy defects, therefore, no Pb^0^ was detected. The use of methylammonium triiodide (MAI_3_) as an additive also effectively mitigated the formation of Pb^0^ by reacting with it (Figure 12f).^7^ The incorporation of certain azaadamantane-based molecular additives effectively suppressed Pb^0^ formation and improved the stability of the perovskite films under light and heat exposure conditions. These modifiers likely interact with defect sites on the perovskite surface, preventing the formation of Pb^0^ and other degradation products.^93^ 2-mercaptonicotinic acid (2-MNA) was proposed as a novel chelating agent for managing excess PbI_2_ in PSCs.^94^ The multifunctional interaction of 2-MNA with PbI_2_, facilitated by its carbonyl, sulfhydryl, and pyridinyl groups, effectively prevents the aggregation of PbI_2_ residues and promotes their orderly distribution at grain boundaries. The chelating coordination of 2-MNA with Pb^2+^ ions of the [PbX_6_]^4–^ octahedron hinders the degradation of PbI_2_-rich perovskites, leading to a suppression in Pb^0^ and I_2_ species formation under environmental stressors. Similarly, P=O/C–O groups of the difluoromethyl phosphonic acid diethyl ester (2F-PAE) additive can interact with uncoordinated Pb^2+^ and prevent the formation of Pb^0^.^95^ The incorporation of low concentrations of methylamide as an additive was found to mitigate Pb^0^ defect formation and improve PL lifetimes, indicating effective suppression of Pb^0^ content by optimal concentrations of amido Pb impurities.^60^ Suppression of Pb^0^ content can be through preventing the generation of Pb dimers, which are intermediates in the mechanism of Pb^0^ formation.^96,97^ 4-*tert*-Butyl-1-(ethoxycarbonylmethoxy)­thiacalix­[4]­arene (tBuTCA) as an additive, with its negatively charged cavities, interacts with Pb^2+^ ions in the perovskite film, preventing the generation of Pb dimers and thereby inhibiting Pb^0^ formation.

The additive strategy was also explored for a quasi-2D perovskite composition namely PEA_*x*_PA_2–*x*_(CsPbBr_3_)_*n*−1_PbBr_4_ (0 ≤ *x* < 2) by employing a bifunctional ligand, 4-(2-aminoethyl)­benzoic acid (ABA), into the perovskite film.^98^ The ABA ligand interacts with the Pb–Br framework, leading to passivation of the bromide vacancies in the perovskite lattice, thus effectively suppressing the formation of Pb^0^. Another additive for suppressing the formation of Pb^0^ is poly­(methyl methacrylate) (PMMA). However, PMMA was introduced not in the precursor solution but instead in the chlorobenzene/toluene antisolvent that was injected during the spin coating of the mixed cation (MA/FA) mixed halide (I/Br) perovskite films.^99^ The reduction in the Pb^0^ signal upon using PMMA was suggested to be due to more stoichiometric iodide content.

The incorporation of additives into the perovskite precursor solutions represents a highly promising approach to suppress the formation of Pb^0^ in perovskite materials. As discussed above, additives like hypophosphorous acid, BQ, and F4TCNQ interact with undercoordinated Pb^2+^ offering a high precision approach to defect passivation. These works indicate the importance of chemical engineering at the precursor level in the suppression of Pb^0^. The development of novel additives tailored to the suppression of Pb^0^ and other degradation pathways will be a critical route for improving the stability of perovskite-based devices. Furthermore, the exploration of a combination of additives that target different degradation mechanisms and investigating their synergetic effects on perovskite materials could hold a significant potential to create more robust perovskite compositions with minimal defects and higher performance.

#### Metal Cation Additives

The addition of metal cations and their complexes has proven effective in stabilizing perovskite structures and mitigating defects such as Pb^0^. Potassium fluoride (KF) incorporation into the perovskite precursor solution was confirmed by XPS analysis to reduce Pb^0^ content.^100^ It was hypothesized that K^+^ ions form bonds with the halide ions in the KF-alloyed perovskites. This interaction is expected to suppress the decomposition of PbI_2_ and consequently reduce the production of Pb^0^ impurities, thereby enhancing the stability of the perovskite material. In another study, the formation of Pb^0^ in DMAPbI_3_ single crystals was suppressed by Cu ion implantation (Figure 13a).^101^ Cu ions interact with unbonded Pb^0^ during the implantation process, thus, preventing the aggregation or further reduction of Pb^2+^ ions to Pb^0^. Introducing Ni^2+^ ions during the film fabrication process helps generate porous PbI_2_ films due to differences in solubility between NiCl_2_ and PbI_2_.^102^ This facilitates MAI penetration and leads to larger perovskite grains. Ni^2+^ ions effectively passivate PbI_3_^–^ defects and reduce the generation of Pb^0^. The strong electron interaction between Ni^2+^ ions, MA cations, and the octahedral [PbI_6_]^4–^ structure helps stabilize the film and suppress defect formation. The Ni^2+^ ions chemisorb at grain boundaries, effectively passivating the sites and suppressing defect formation, leading to better film quality and higher device performance.

**Figure 13:**
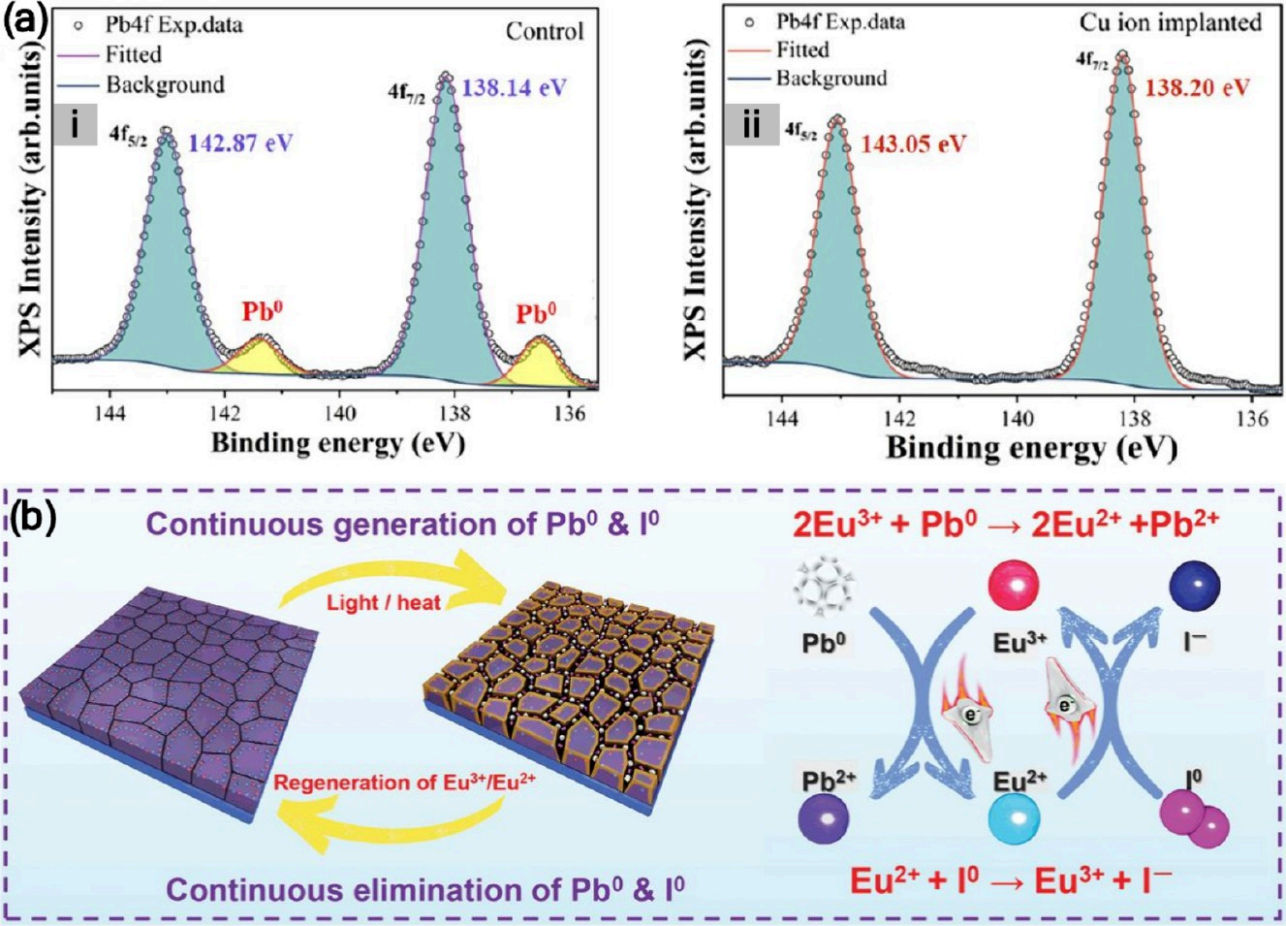
(a) XPS spectra of Pb 4f for (i) control and (ii) Cu ion implanted DMAPbI_3_ PSCs. Reproduced with permission from ref 101. Copyright 2023 Elsevier. (b) Schematic illustration of the proposed mechanism involving the cyclic elimination of Pb^0^ and I^0^ defects and regeneration of Eu^3+^–Eu^2+^ redox pair. Reproduced with permission from ref 4. Copyright 2019 the authors of ref 4, exclusive licensee American Association for the Advancement of Science.

#### Redox-Active Additives

Another innovative approach involves the Eu^3+^–Eu^2+^ ion redox shuttle, which selectively oxidize Pb^0^ and reduce I^0^ defects simultaneously in a cyclic transition (Figure 13b).^4^ In this cyclic redox transition, Pb^0^ defects are oxidized by Eu^3+^, while I^0^ defects are simultaneously reduced by Eu^2+^ as demonstrated in the two chemical reactions below:

$$2{\mathrm{Eu}}^{3+}+{\mathrm{Pb}}^{0}\to 2{\mathrm{Eu}}^{2+}+{\mathrm{Pb}}^{2+}$$

$${\mathrm{Eu}}^{2+}+I^{0}\to {\mathrm{Eu}}^{3+}+I^{-}$$

The Eu^3+^–Eu^2+^ pair remains nonvolatile and maintains suitable redox potentials for this cyclic transition, allowing for continuous defects elimination during device operation. The Eu complex as a redox shuttle for the elimination of Pb^0^ was also utilized later by other researchers.^103,104^

Another redox shuttle for the suppression of Pb^0^ is the iodide/triiodide (I^–^/I_3_^–^) redox shuttle.^105^ Using I_2_ and 1-butyl-3-methylimidazolium iodide in mixed DMSO/DMF solution, this shuttle was introduced as an additive to continuously passivate Pb^0^ and I^0^ defects in perovskite films. The mechanism by which the I^–^/I_3_^–^ redox shuttle suppresses Pb^0^ formation in perovskite films involves of oxidation–reduction reactions that occur between the redox shuttle species and the Pb^0^ and I^0^ defects as follows:

$${\mathrm{Pb}}^{0}+{I_{3}}^{-}\to {\mathrm{PbI}}_{2}+I^{-}$$

$$2I^{0}+I^{-}\to {I_{3}}^{-}$$

Mesoporous MIL-101­(Cr) was explored as a support for hosting Keggin-type polyoxometalates (POMs) CoW_12_ to create a stable POM-based material, CoW_12_@MIL-101­(Cr), aiming at eliminating Pb^0^ and passivating Pb^2+^ ions.^106^ The oxidative ability of CoW_12_ is enhanced via the interaction with the MOF framework of MIL-101­(Cr), allowing for controlled elimination of Pb^0^ defects without the need for oxidizing I^–^ anions. The strong interaction between CoW12@MIL-101­(Cr) and uncoordinated Pb^2+^ ions results in doped perovskite films with uniform morphology and enhanced light absorption.

The incorporation of metal cation additives into the perovskite precursor solution represent an advanced approach to stabilizing perovskite structures and reducing defect-related degradation. In particular, the Eu^3+^–Eu^2+^ pair introduces a dynamic method for continuously managing Pb^0^ and I^0^ defects. This approach is promising because it provides a mechanism for ongoing defect correction during device operation, which could significantly extend the long-term stability and efficiency of perovskite solar cells and other optoelectronic devices. Future research on stabilizing perovskite structure and suppressing Pb^0^ formation will depend on the continued exploration of chemical interactions and dynamic defect management strategies. These approaches not only address the immediate challenge of Pb^0^ formation but also open up new possibilities for enhancing the overall stability and efficiency of perovskite-based technologies. However, care must be taken to avoid introducing secondary phases or unwanted deep-level traps. Hence, dopant incorporation must be precisely controlled, and codoping strategies may be required to maintain charge neutrality and lattice coherence.

#### Post-treatment and Interlayers

In a post-treatment strategy, 3-hydroxypyridine was utilized to suppress the Pb^0^ signal in XPS.^107^ The fabricated MAPbI_3_ films were immersed in 3-hydroxypyridine solutions, which suppressed the formation of Pb^0^, possibly due to surface passivation minimizing iodine loss under X-rays. Post-treatment of MAPbI_3_ films by casting an isopropanol solution of 4-fluorophenethylammonium iodide was also recently reported to reduce the Pb^0^ content in the perovskite films.^108^ Strategies such as reducing excess PbI_2_ in perovskite films by employing low-dimensional perovskites *n*-butylammonium bromide (BABr) as inhibitors can effectively restrain the formation of Pb^0^.^59^ The post-treatment of the triple CsFAMA perovskite with a layer of BABr formed a Ruddlesden–Popper (RP) phase as confirmed by XRD, and more importantly eliminated the excess PbI_2_.

The formation of Pb^0^ within the RP perovskites like phenylethylammonium lead iodide (PEA_2_PbI_4_) can be suppressed through various methods, including molecular surface passivation.^109^ In this study, the deposition of a strong molecular acceptor, such as 2,20-(perfluoronaphthalene-2,6-diylidene) dimalononitrile (F6-TCNNQ), on top of the perovskite layer effectively eliminated existing Pb^0^ defects and prevented the formation of new ones (Figure 14a). This passivation mechanism involves electron transfer from Pb^0^ to the molecular acceptor, leading to the oxidation of Pb^0^ to Pb^2+^ and stabilizing the perovskite film. Additionally, molecular surface passivation helps cover the reactive terrace edges of the perovskite film, where Pb^0^ formation is initiated, thereby enhancing the photostability of the material.

**Figure 14:**
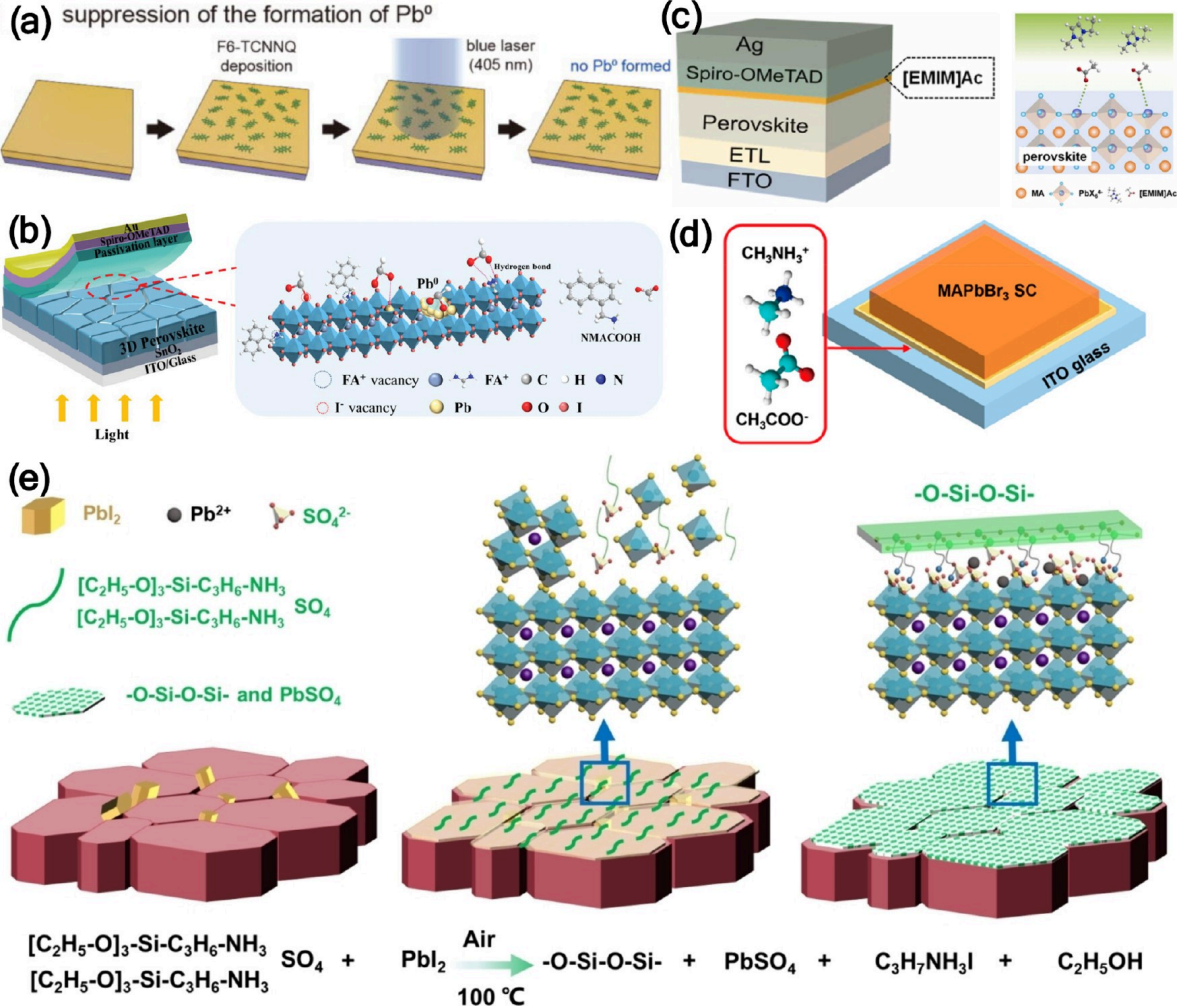
(a) Schematic overview of the sample preparation method in which the organic molecular dopant F6-TCNNQ was deposited on nonirradiated PEA_2_PbI_4_ to suppress subsequent Pb^0^ formation under irradiation. Reproduced from ref 109. CC BY 4.0. (b) Schematic representation of the PSC structure and the proposed passivation mechanism of NMACOOH. Reproduced with permission from ref 110. Copyright 2023 Wiley-VCH. (c) Schematic diagram of the PSC structure incorporating the ionic liquid [EMIM]Ac between the perovskite film and the hole transport layer along with schematic illustration of [EMIM]­Ac-treated perovskite surface. Reproduced with permission from ref 111. Copyright 2023 Elsevier. (d) Schematic illustration of a MAPbBr_3_ SC integrated onto an ITO glass substrate using methylammonium acetate. Reproduced from ref 112. Copyright 2023 American Chemical Society. (e) Illustration of a gradient lead sulfate–silica bilayer formed on the surface of PbI_2_ with KHSO. Reproduced with permission from ref 114. Copyright 2023 Wiley-VCH.

A new approach for suppressing the formation of Pb^0^ in PSCs is based on using a nonhalide ionic salt, 1-naphthylmethylammonium formate (NMACOOH), for surface passivation of perovskite films (Figure 14b).^110^ Traditionally, alkylammonium halides have been used for passivation to suppress nonradiative recombination in PSCs. However, these compounds can exhibit high surface reactivity, leading to undesirable transformations, such as the formation of 2D perovskite phases, under working conditions. This limits their passivation effectiveness and negatively impacts charge transport in the devices. In contrast, NMACOOH post-treatment hinders the formation of 2D perovskite phases and forms a stable PbI_2_–NMACOOH adduct on the perovskite surface. This passivation effect is attributed to the coordination ability of the formate anion (HCOO^–^) with Pb^2+^ ions on the perovskite surface. Specifically, Pb^2+^ ions preferentially coordinate with HCOO^–^, preventing the formation of Pb–I octahedrons and inhibiting the transformation of the passivating agent into 2D perovskite. As a result, the perovskite film remains in the desired 3D phase, enhancing its stability and passivation effectiveness. Moreover, the NMACOOH-treated perovskite film demonstrates improved thermal stability, as evidenced by the inhibition of Pb^0^ formation even under elevated temperatures. This stability is attributed to the interaction between HCOO^–^ and residual PbI_2_ on the perovskite surface, which suppresses the formation of Pb^0^ and reduces nonradiative recombination losses.

The introduction of the 1-ethyl-3-methylimidazolium acetate ([EMIM]­Ac) ionic liquid through spin coating as an interlayer between the perovskite (MAPbI_2.85_Br_0.15_) film and Spiro-OMeTAD effectively suppressed the formation of Pb^0^ by coordinating with uncoordinated Pb^2+^ on the perovskite surface (Figure 14c).^111^ Similarly, methylammonium acetate (MAAc) can passivate Pb^0^ in MAPbBr_3_ SCs by forming a new interfacial layer between perovskite and substrate (Figure 14d).^112^ This layer mediated a strong bond between the perovskite crystal and the ITO substrate. Another explored interlayer is the hyperbranched polyamide-amine-based polymers (HBPs) that was introduced between the electron transport layer (SnO_2_) and the perovskite active layer resulting in strong adhesion at the interface and the prevention of Pb^0^ formation.^113^

Inserting interlayers like graphene between the metal electrodes and the halide perovskite could prevent direct contact and inhibit the formation of Pb^0^.^72^ Adding carbon dots (CDs) to the perovskite film suppresses the formation of Pb^0^.^46^ CDs selectively aggregate around and over the surface of perovskite crystals, forming a wrapping layer. This CD layer acts as a barrier, preventing the evaporation of the organic cations from the perovskite surface and grain boundaries during thermal annealing. Another strategy to suppress the formation of Pb^0^, coming from excess PbI_2_, is through surface reconstruction to convert the excess PbI_2_ into a gradient lead sulfate-silica bilayer (Figure 14e).^114^ This conversion results in not only suppression of Pb^0^ but also in a homogeneous layer that enhances the stability of the perovskite film and reduces interfacial charge transfer barriers in PSCs devices. The bilayer is formed through dripping ((C_2_H_5_O)_3_Si­(CH_2_)_3_NH_3_)_2_SO_4_ on the perovskite surface.

The integration of heteroatom-substituted organic molecules as hole transport interlayers in PSCs can both suppress Pb^0^ formation and enhance the charge transport properties. In detail, heteroatom (O, N) substituted organic hole transporting layers namely **YZ22**, **PhI-TPA**, and **BTZI-TPA** were utilized to inhibit the Pb^0^ formation at the perovskite/HTM interface.^115,116^ As shown in Figure 15a, molecule **YZ22** has ability to passivate the perovskite surface by forming coordination bonds between the N atoms of the central phenanthroline unit and the undercoordinated Pb^2+^ ion of the perovskite. In contrast, molecule **YZ18** containing central phenanthrene with no N atoms, fails to suppress the formation of Pb^0^ perovskites surface. Molecules **PhI-TPA** and **BTZI-TPA** passivate the perovskite surface by forming coordination bonds between O and N atoms of their central pyrrolidinedione and thiadiazol units, respectively, with the undercoordinated Pb^2+^ ion at the perovskite surface. These strongly coordinated N···Pb^2+^ and O···Pb^2+^ interactions suppress the decomposition of Pb^2+^ to Pb^0^ (Figure. [Fig fig15]a). The XPS analysis of the perovskite films treated with the different organic molecules are shown in Figure 15b,c. Recently, it was also demonstrated that the use of thick polymer-based HTLs (e.g., PTAA ∼ 35 nm) instead of the thin MeO-2PACz could block the electron injection at the ITO/HTL interface, reducing the chance for Pb^2+^ reduction to Pb^0^.^56^

**Figure 15:**
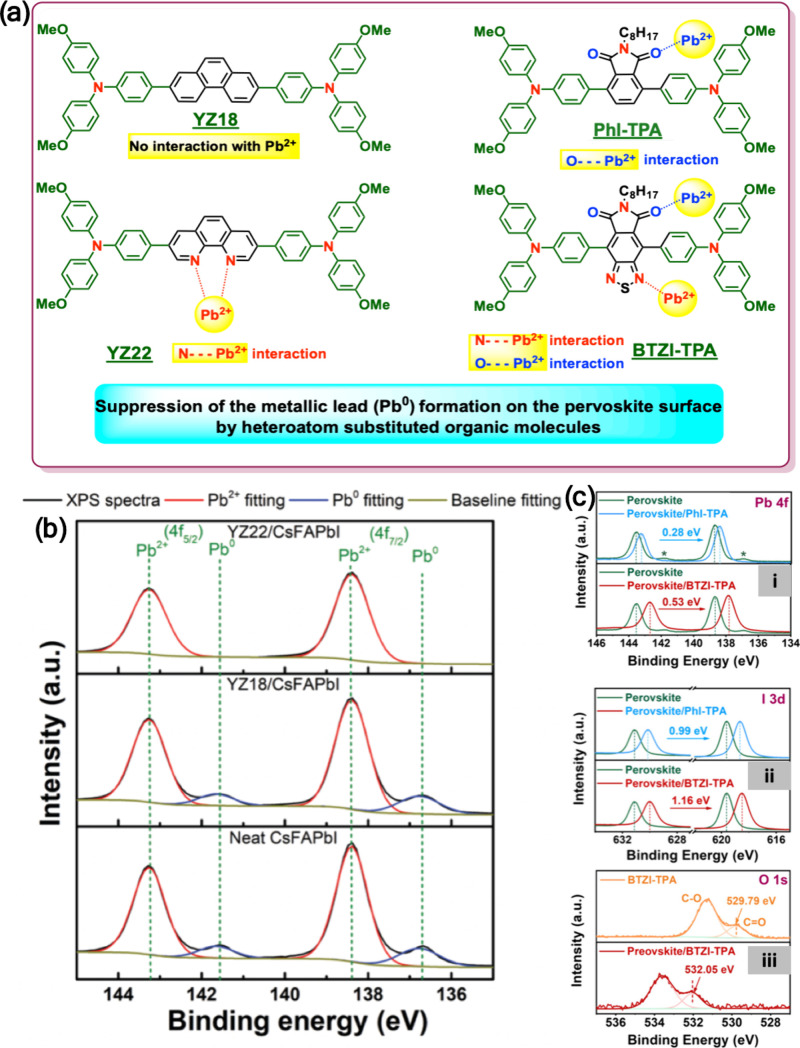
(a) Molecular structures of hole-transporting passivators and their interaction with Pb^2+^ ions. (b) Pb 4f core-level XPS spectra of CsFAPbI with a stack comprising **YZ18** and a stack comprising **YZ22** on the perovskite. Adapted with permission from ref 115. Copyright 2020 Royal Society of Chemistry. (c) XPS spectra of (i) Pb 4f and (ii) I 3d for the control, **PhI-TPA-** and **BTZI**-modified perovskite films and (iii) XPS spectra of N 1s and O 1s for the **BTZI-TPA** and perovskite/**BTZI-TPA** film. Adapted from ref 116. Copyright 2022 American Chemical Society.

Future research could explore the development of new passivation molecules that can suppress the defects even under extreme conditions such as intense light exposure and elevated temperatures. The use of interlayers, such as CDs and gradient lead sulfate-silica bilayers, exemplifies the trend toward integrating multifunctional layers that protect against degradation while optimizing device performance. The focus on interlayers that can dynamically manage surface defects and inhibit Pb^0^ formation is expected to drive further innovations. Particularly, the development of interlayers involving heteroatom-substituted organic hole transport materials will be crucial for improving the scalability and commercial viability of perovskite-based devices. These strategies highlight the importance of a full approach to perovskite material design, involving not only the suppression of defects but also the optimization of interfacial properties and charge transport mechanisms. Future work should target scalable interlayer deposition methods (e.g., blade coating, ALD, or inkjet printing) and multifunctional designs that simultaneously enhance charge transport, thermal stability, and Pb^0^ suppression.

#### Light Modulation, Bandpass Filters, Applied Voltage, and Pulsed UV Laser Treatment

Pb^0^ formation, while known to occur due to the photodecomposition of PbI_2_, it was recently reported that this takes place only under intense illumination (1 sun).^117^ Under such conditions, the iodine in the solution can be oxidized into I_3_^–^, which then leads to the formation of Pb^0^. In contrast, under low-intensity illumination (0.01 sun), this decomposition of PbI_2_ and subsequent formation of Pb^0^ was not observed.

Strategies to suppress Pb^0^ formation include the use of wavelength-selective bandpass filters, which selectively transmit light within specific wavelength ranges.^30^ Reduced Pb^0^ formation is achieved by blocking light within the absorption range of PbI_2_ domains.

The formation of Pb^0^ in MAPbI_3_ perovskite films involves complex processes influenced by various factors including applied voltage.^118^ By applying an extraction voltage to the device contacts, the photogenerated charge carriers are effectively removed from the perovskite film. This extraction prevents the accumulation of charge carriers and thereby suppresses the decomposition of the perovskite and the formation of Pb^0^. Hence, under operational conditions where charge carriers are continuously extracted (such as when the solar cell is connected to a load), the formation of Pb^0^ is minimized compared to open-circuit conditions.

Recently, a pulsed UV laser treatment of [(FAPbI_3_)_0.87_­(MAPbBr_3_)_0.13_]_0.92_­[CsPbI_3_]_0.08_ was suggested to effectively eliminate the Pb^0^ defects from the film’s surface as evidenced by XPS.^119^ This noncontact method makes it a simple to passivate surface defects without introducing any additives that may introduce chemical complexity into the perovskite films.

Effective strategies for suppressing Pb^0^ formation include wavelength-selective filters, extraction voltages to minimize charge accumulation, and pulsed UV laser treatments for noninvasive defect passivation. These methods underline the importance of operational passivation where degradation can be dynamically managed through control of the working environment. Combining these approaches can significantly enhance perovskite device stability and efficiency. Future research could focus on optimizing these strategies and exploring the synergy between material engineering and operational conditions for large-scale fabrication of stable perovskite devices.

## Interesting Advantage of Pb^0^

In contrast to most works reporting on the bane of the degradation of halide perovskite and the formation of Pb^0^, it has been demonstrated that the degradation of MAPbBr_3_ under an electron beam leading to the formation of Pb^0^ is, in fact, beneficial for photodetector applications.^120^ MAPbBr_3_ microplates irradiated with low energy (10–20 keV) electron beam displayed PL quenching due to the formation of Pb^0^ at the surface of the microplates. Nevertheless, when these irradiated MAPbBr_3_ microplates were utilized as an active layer in photodetectors, the photocurrent density increased by 217% compared to the pristine microplates. This phenomenon was attributed to the Pb^0^ ability to collect photogenerated electrons, hence reducing the recombination process and improving the concentration of the photogenerated electrons. Furthermore, while Pb^0^ forms in MAPbI_3_ films upon immersion in toluene, especially when under illumination, this also results in increased ionic conductivity of the films.^71^ This was suggested to be due to the generation of more mobile iodine vacancies. Future studies should seek to identify and optimize such beneficial roles rather than exclusively viewing Pb^0^ as a detrimental byproduct.

## Pb^0^ in Perovskite Nanomaterials

Using XPS, Pb^0^ was also detected in perovskite nanomaterials such as MAPbBr_3_ nanocrystals (NCs) and CsPbX_3_ nanoplatelets (NPLs), and it was suggested to be electron beam-induced.^121–125^ The origin of Pb^0^ formation in CsPbBr_3_ quantum dots (QDs) is linked to their degradation, causing Pb leakage and structural instability.^126^ This lead leakage results in Pb^0^ nanoparticles (NPs) aggregation within the QDs. In detail, in situ STEM analysis was conducted in which additional diffraction spots that do not belong to the QDs were observed, indicating the presence of an impurity attached to the QDs. Combined high-resolution STEM with EELS identified the impurity as Pb^0^ (Figure 16a). Interestingly, the formation of Pb^0^ NPs was observed to coincide with the formation of an RP phase (Cs_2_PbBr_4_) separated from CsPbBr_3_ by a phase boundary. The decomposition of both CsPbBrI_2_ and CsPbBr_2_Cl NCs under continuous X-ray illumination showed the formation of Pb^0^ gradually over time, with its intensity increasing with prolonged exposure.^127^ Along with Pb^0^ formation, the evolution of I_2_ or Cl_2_, depending on the halide composition of the perovskite was detected. In both cases, the decomposition process was initiated by a contraction of the atomic lattice triggering the decay of the NCs.

**Figure 16:**
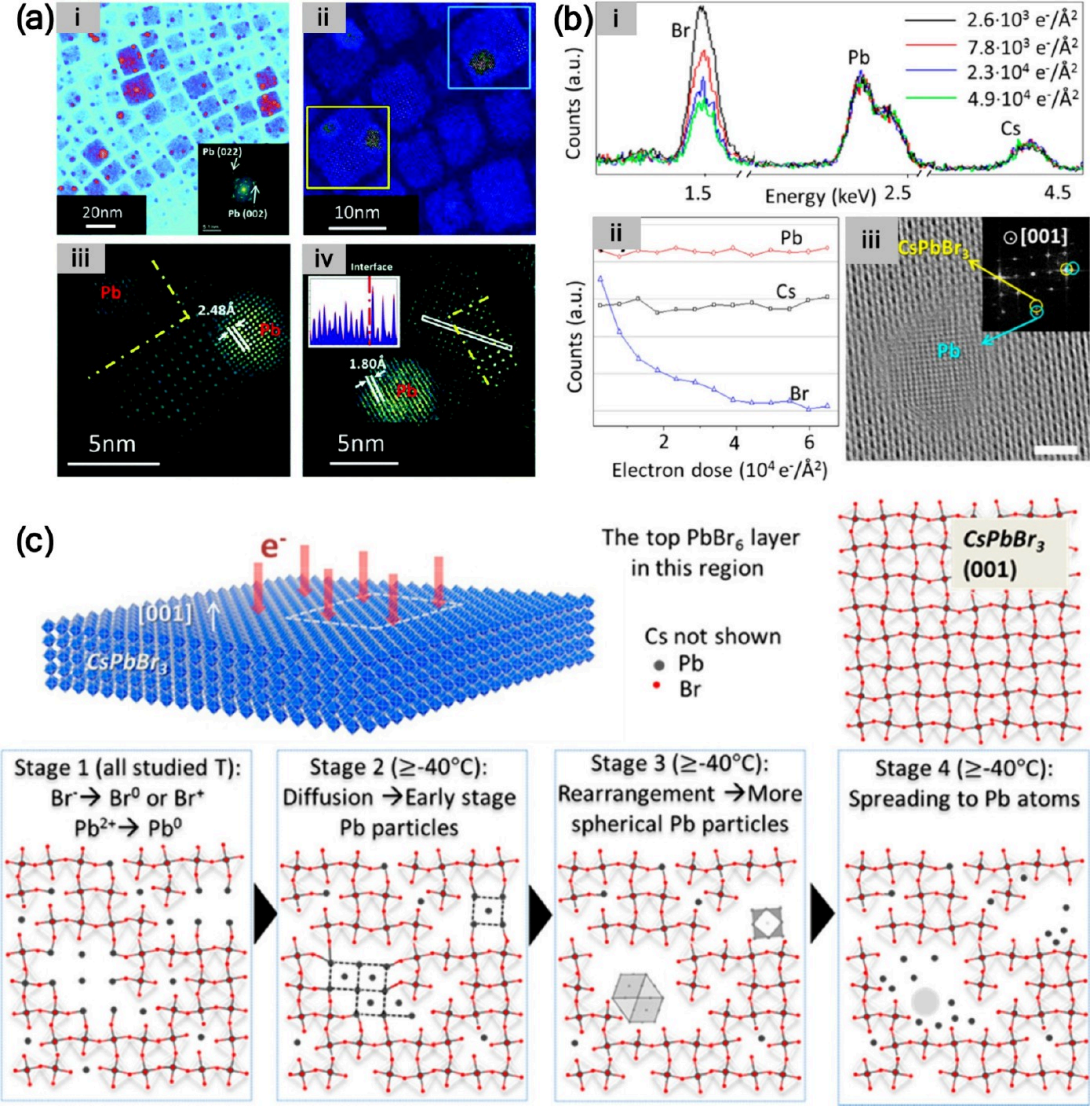
(a) (i) Low-magnification HAADF image of partially degraded CsPbBr_3_ QDs. The inset shows the diffractogram of the entire imaging area. The labeled arrow points to the diffraction spots for Pb^0^. (ii) Atomic-resolution HAADF image of a selected region in (i), with boxes highlighting areas with Pb clusters. (iii) Atomic-resolution HAADF image of a single CsPbBr_3_ QD with a Pb cluster and a distinct phase separated by a yellow dashed line. (iv) Atomic-resolution HAADF image of another CsPbBr_3_ QD. The inset shows the intensity line profile of the yellow rectangle region across a phase boundary. Reproduced with permission from ref 126. Copyright 2020 Royal Society of Chemistry. (b) Electron-beam-induced Br desorption and Pb NPs formation during electron irradiation of 3 nm thick CsPbBr_3_ NSs at RT: (i) EDS spectra showing elemental changes at increasing electron doses; (ii) plot of the net integrated peak intensity for Cs, Pb, and Br as a function of the electron dose from STEM-EDS analysis; (iii) HRTEM of a Pb NP formed on CsPbBr_3_ NS, with both CsPbBr_3_ and Pb oriented along the [001] zone axis (scale bar: 2 nm), and (inset) Fast Fourier Transform (FFT) with the spots for CsPbBr_3_ 220 and Pb 200 circled. (c) Schematics diagrams illustrating the structural evolution of an irradiated area in a CsPbBr_3_ NS oriented along the [001] zone axis. Reproduced from ref 128. Copyright 2017 American Chemical Society.

A study on the mechanism of Pb^0^ formation in CsPbBr_3_ NCs revealed a radiolysis process in CsPbBr_3_ NCs, involving the electron beam-induced desorption of some of Br atoms and the reduction of a portion of Pb^2+^ ions to Pb^0^ (Figure 16b).^128^ Afterward, Pb^0^ atoms exhibit diffusion and aggregation, which gives rise to distinct high-contrast particles under TEM imaging (Figure 16b). Initially, the formed Pb^0^ clusters are epitaxially bound to the parent CsPbBr_3_ lattice; however, upon further irradiation, they transform into nonepitaxially bound Pb crystals. This progression leads to local amorphization, resulting in the CsPbBr_3_ lattice being disassembled (Figure 16c). Comparative analysis across CsPbBr_3_ NCs with varied shapes and sizes indicated that damage is particularly pronounced at the corners and edges of the surface. This is attributed to a lower diffusion barrier for Pb^0^ on the surface than inside the crystal, along with a larger proportion of undercoordinated atoms in these regions.

The formation of Pb^0^ in the 0D Cs_4_PbBr_6_ ^129,130^ NCs dispersed in a solvent was also investigated by liquid cell transmission electron microscopy (LCTEM).^131^ Pb^0^ NPs were formed under an electron beam in the areas of the solution where no Cs_4_PbBr_6_ NCs are seen, suggesting that Cs_4_PbBr_6_ NCs are not the precursor for Pb^0^. Instead, Pb^0^ formation was attributed to the presence of Pb^2+^-containing species in solution, such as Pb­(II) bromide/oleate species. The reduction of Pb^2+^ ions leads to the aggregation of Pb^0^ atoms into small particles, which nucleate and grow into Pb^0^ NPs. This mechanism follows an autocatalytic surface growth process, where Pb^0^ nuclei consume available Pb^2+^ ions, transitioning to the nanoparticle growth stage.

In MAPbBr_3_ NCs, the origin of Pb^0^ is the reduction of Pb^2+^ ions at the surface of MAPbBr_3_ NCs, facilitated by the presence of Br^–^ vacancies at the corners of the PbBr_6_^4–^ octahedra.^132^ Nevertheless, in the presence of organic ligands, such as n-octylamine (OAm), the amine end group can be protonated to an ammonium cation (R-NH_3_^+^), which coordinates with PbBr_6_^4–^ octahedra and prevents the conversion of Pb^2+^ to Pb^0^. Suppression of Pb^0^ was also reported in CsPb­(Cl/Br)_3_ NCs by treating the NCs with hexahydrated nickel nitrate.^133^ The introduced nitrate ions stripped off labile lead atoms, uncoordinated Pb^2+^, excess Cs^+^, and a portion of the halides, resulting in perovskite QDs with fewer surface defects. The formation of Pb^0^ can be suppressed or slowed down by modifying the surface ligands of the perovskite NCs. For example, ligand exchange with metal porphyrin derivatives or other stabilizing ligands can alter the decomposition pathway and kinetics, leading to a slower decay rate and reduced formation of Pb^0^. Additionally, optimizing the synthesis conditions and using protective coatings or encapsulation techniques may help mitigate the formation of Pb^0^ and enhance the stability of CsPbX_3_ perovskite NCs under X-ray irradiation. Interestingly, it was recently reported that excess PbI_2_ coordinated with oleate and oleylamine as a passivating agent could improve the PL of the NPLs through reducing the surface Pb^0^ content.^125^

The persistent challenge of Pb^0^ formation in perovskite nanomaterials calls for a deeper understanding of the underlying mechanisms and the development of targeted solutions to enhance the material’s stability. Pb^0^ formation, often induced by factors such as electron beam irradiation and the presence of surface defects, not only degrades the material but also compromises its optoelectronic properties, which are critical for applications in solar cells, LEDs, and other devices. From a broader perspective, addressing this issue requires a multifaceted approach. The role of surface chemistry, particularly the interaction between Pb^2+^ ions and organic or inorganic ligands, must be further explored. For example, the suppression of Pb^0^ through the use of passivating agents like oleylamine highlights the importance of ligand design in controlling surface reactions. Future research should focus on identifying and synthesizing new ligands or surface treatments that can effectively prevent Pb^0^ formation while preserving or enhancing the desired properties of the perovskite nanomaterials. Moreover, understanding the impact of crystal morphology, such as the shape and size of nanocrystals, on Pb^0^ formation could lead to more tailored synthesis approaches that minimize defect sites. Investigating the effect of different halide compositions on the stability of the perovskite lattice under operational conditions could also yield insights into designing more resilient materials. Ultimately, the goal is to develop perovskite nanomaterials that not only exhibit high performance but also maintain their structural integrity over time, even under harsh conditions. Beyond materials, the development of advanced in situ characterization techniques (e.g., operando TEM or photothermal IR spectroscopy) will be essential to unravel the kinetics and triggers of Pb^0^ formation in NCs. By addressing the challenge of Pb^0^ formation, the field can move closer to realizing the full potential of perovskite-based technologies in commercial applications, paving the way for more durable and reliable optoelectronic devices.

## Conclusion and Outlook

The formation of Pb^0^ in halide perovskites represents a critical bottleneck in the path toward the commercial deployment of perovskite-based devices. This process, whether initiated by external environmental stressors like X-ray irradiation, visible light exposure, elevated temperatures, or internal material instabilities stemming from synthesis and fabrication protocols, introduces deep trap states and accelerates structural degradation. These defects compromise charge carrier lifetimes, induce nonradiative recombination, and ultimately reduce the operational efficiency and lifespan of devices.

What emerges clearly is that Pb^0^ formation is a multifactorial phenomenon, influenced not only by isolated triggers but also by synergistic interactions among thermal, and chemical stimuli. For instance, illumination under vacuum or in the presence of moisture can accelerate Pb^0^ formation via complex pathways involving iodide vacancy generation and charge accumulation. Heating plays a dual role in accelerating the decomposition of halide perovskites and facilitating the reduction of Pb^2+^ to Pb^0^ through both thermodynamic and kinetic pathways. Thermodynamically, elevated temperatures destabilize the perovskite lattice by weakening the ionic bonds between the A-site cations (e.g., MA, FA) and the halide framework, promoting the release of volatile species such as MAI and HI, and leading to the formation of PbX_2_. These PbX_2_ decomposition products are less stable and are more susceptible to further reduction to Pb^0^. Kinetically, heating enhances ion mobility and halide vacancy generation, which can trap electrons and facilitate the reduction of Pb^2+^. Studies have also shown that the mobility of Pb^0^ atoms increases with temperature, accelerating their diffusion and aggregation into larger clusters. Notably, nitrogen-rich environments significantly suppress the photo- and X-ray-induced formation of Pb^0^, highlighting the critical role of ambient species such as O_2_ and H_2_O in facilitating halide vacancy formation and Pb^2+^ reduction. These findings underline the need for inert encapsulation strategies and support a broader mechanistic understanding of how environmental conditions modulate perovskite stability pathways. Furthermore, the varying time constants of degradation under different stressors highlight the importance of balancing illumination intensity, environmental control, and thermal exposure in device stability design.

Efforts to suppress Pb^0^ formation include compositional tuning, the inclusion of redox-stable additives, and the development of interfacial passivation layers. Chemically, mixed A-site cations such as Cs^+^ and FA enhance structural stability by minimizing volatile degradation products like NH_3_ and CH_3_I (as in the case of MA) and by reducing defect densities. Redox-stable additives such as Eu^3+^ not only reduce intrinsic defect concentrations but may also act as alternative redox centers, intercepting photogenerated electrons before they reduce Pb^2+^ to Pb^0^. Similarly, surface passivation strategies work by chemically coordinating undercoordinated Pb^2+^ or halide vacancies with Lewis base ligands, thereby stabilizing the surface against electron accumulation. However, these strategies are often highly specific, with their effectiveness varying across device architecture, environmental exposure, and fabrication route. Hence, generalized solutions are rare, and future progress will depend on understanding the interdependencies between compositional, morphological, and environmental factors. Moreover, techniques that emphasize reversibility, rather than prevention, represent a promising frontier that aligns with the broader trend of self-healing materials.

While this review emphasizes the great efforts made in identifying the conditions and compositions that accelerate Pb^0^ formation, a systematic understanding of the relative impact and coupling of these factors remains underdeveloped. For example, the simultaneous effect of X-ray exposure and electrical bias during medical imaging, or the interaction of UV light with moisture during outdoor PV operation, remains to be quantified. Future work must prioritize multifactorial aging studies and operando characterization techniques to reveal degradation mechanisms as they occur in real-world environments. Beyond highlighting the critical degradation pathways in halide perovskites, future studies should also aim to answer targeted mechanistic questions using advanced in situ and operando techniques. For instance, what are the real-time kinetics of Pb^0^ formation under varying environmental conditions such as light intensity, and temperature? How do defect migration, ion segregation, and surface reconstruction contribute to Pb^0^ accumulation at different interfaces? What are the atomistic differences in degradation mechanisms between triple-cation and single-cation systems, and how do they translate to improved long-term stability? Addressing such questions will require time-resolved spectroscopies, environmental TEM/XPS, and computational modeling to deconvolute competing thermodynamic and kinetic factors. Standardized degradation protocols and comparative studies across compositions and architectures will be essential to translate laboratory-scale findings into durable real-world optoelectronic applications.

To this end, in Table 1, we categorize the key environmental and synthetic factors contributing to Pb^0^ formation and maps them to suppression strategies that range from chemical additives to fabrication optimizations. This serves as both a reference point for researchers and a roadmap for future work aiming to develop truly stable perovskite systems.

**Table 1:** Summary of Factors Contributing to Pb^0^ Formation in Halide Perovskites and Their Proposed Prevention Strategies

factors contributing to Pb^0^ formation	contribution mechanism	prevention methods/future outlook
high-energy radiation (X-ray, γ ray)	halide loss and bond cleavage	introduce:
		• crystal lattice stabilizing additives (e.g., alkali and alkaline-earth metal salts).
		• surface and grain boundary passivating agents (e.g., organic ligands and ammonium salts)
		• radical scavengers (e.g., fullerenes, phenyl-based additives, thiols).
		• radiation-resistant encapsulation polymers (e.g., PMMA, PVDF) or inorganic encapsulation (e.g., Al_2_O_3_, TiO_2_).
visible light illumination	halide vacancies and photogenerated electron trapping	introduce:
		• photostabilizers (organic ammonium salts, benzophenones, fullerenes).
		• UV/vis-blocking polymeric coatings (e.g., PMMA, PC) or inorganic coatings (e.g., ZnO, TiO_2_).
thermal effects	thermally induced ion migration and A-site organic cation decomposition	• precise control of annealing temperature and time
		• use solvent engineering for controlled crystallization
		• low-temperature processing techniques
		• thermally stable additives (alkali cations, polymeric passivation)
electron beam exposure (SEM/TEM)	surface atom halide desorption and bond rupture	introduce:
		• beam-stable passivation layers (e.g., amorphous carbon coatings, thiols)
		• protective conductive coatings (e.g., carbon, gold)
ion beam exposure	ion sputtering	introduce:
		• protective inorganic capping layers (e.g., ZnO, TiO_2_, Al_2_O_3_)
moisture (H_2_O) exposure	hydrolysis of A–X bonds	• hydrophobic ligand passivation (e.g., organic ammonium salts, organic lignads, fluorinated alkylamines).
		• encapsulation with moisture barriers (e.g., ALD Al_2_O_3_, polymers).
applied voltage	electric-field-driven ion migration and interface reactions	• interface engineering (ETL/HTL and perovskite).
		• use thick polymer-based HTLs (e.g., PTAA ∼35 nm)
		• employing stable electron/hole transport layers (e.g., SnO_2_, NiO_*x*_, MoO_3_, SAMs)
		• use ion-migration-reducing additives (e.g., alkali metal cations, large organoammonium cations)
excess PbX_2_	Residual PbX_2_ as Pb^0^ source	• precise stoichiometry control
		• surface passivation using organic additives
precursor solution chemistry and composition	solvent coordination	• optimize solvent selection
		• use stabilizing additives (Lewis bases, amines, thiols)
perovskite composition (A, B, and X-site ions)	intrinsic instability (e.g., MA, I^–^ rich)	• utilize intrinsically stable compositions (FA/Cs-based)
		• introduce stable metal cations (K^+^, Rb^+^, Sr^2+^, Zn^2+^)
fabrication technique (spin-coating, vapor deposition)	poor crystallinity and uncontrolled nucleation	• refine processing methods (spin speed, deposition rate)
		• employ solvent-engineering methods
		• use vapor-phase or antisolvent methods for dense, uniform films
		• slow crystallization techniques
interaction with metal contacts (electrodes)	metal–halide reactions, ion diffusion	• apply barrier/interface layers (SnO_2_, TiO_2_, MoO_3_)
		• robust interlayers/barriers to prevent ion migration

Finally, it is important to highlight the reliability of using techniques such as XPS, XRD, and TEM to identify Pb^0^ formation, given that these methods themselves may induce degradation. While it is well recognized that halide perovskites are sensitive to X-ray and electron beams, several studies have demonstrated that with careful experimental design and proper controls, these tools can still provide reliable insights into the presence and distribution of Pb^0^. In the case of X-ray photoelectron spectroscopy (XPS), beam-induced damage can be minimized by employing low-power settings and short acquisition times. To distinguish probe-induced Pb^0^ from photoinduced degradation, comparative measurements between pristine and light-exposed samples are conducted under identical XPS acquisition conditions. If Pb^0^ signals appear only after illumination, this provides compelling evidence of light-induced degradation rather than XPS-induced artifacts. Similarly, XRD can be susceptible to beam-induced effects if scans are conducted at high exposure or over extended durations. Hence, fast scanning modes can limit such damage. TEM is also known to induce significant degradation in halide perovskite NCs, yet, has also proven to be a valuable tool in understanding the mechanistic evolution of Pb^0^. For instance, in the case of CsPbBr_3_ NCs, detailed TEM investigations revealed a radiolysis-driven process where the electron beam induces Br desorption, facilitating the reduction of Pb^2+^ to Pb^0^. The resulting Pb^0^ atoms diffuse and aggregate, initially forming clusters that are epitaxially bound to the perovskite lattice. With continued exposure, these evolve into nonepitaxially bound Pb crystals and eventually lead to local lattice amorphization and structural disintegration. Moreover, comparative analyses across NCs of varied shapes and sizes have shown that the damage is more pronounced at corners and edges, consistent with lower diffusion barriers and higher densities of undercoordinated surface atoms in these regions.

The complete mitigation of Pb^0^ formation would extend their usability into commercial sectors such as solar power, low-dose medical imaging, and flexible displays, where durability and reproducibility are crucial. Through a combination of fundamental mechanistic insight, compositional design, process engineering, and in situ monitoring, the field could overcome this critical obstacle.

## Vocabulary

**Metallic lead (Pb** ^**0**^ **)**: Zero-valent lead that forms either (i) by reduction of Pb^2+^ due to external factors (e.g., light, heat, or electrical bias) or (ii) during crystallization from redox-imbalanced precursor chemistries (e.g., halide-deficient/PbI_2_-rich stoichiometry, reducing solvents/additives, or A-site depletion). Pb^0^ creates charge carrier trap states and degrades device performance.
Undercoordinated Pb: Pb^2+^ sites with fewer than six halogen neighbors (common at surfaces/grain boundaries). These electron-accepting sites are reactive, promote trap formation, and are primary sites for Pb^2+^ to Pb^0^ reduction unless passivated.
**Halogen vacancy (V**_**X**_ **)**: A point defect where a halogen (I, Br, Cl) is missing from its regular lattice site. Halogen vacancies lower the barrier for electron trapping and for Pb^2+^ reduction to Pb^0^. V_X_ is mobile under various conditions (e.g., illumination and heat), enabling defect clustering and ion migration.
Passivation: Chemical treatment that neutralizes electronic defects (e.g., binding undercoordinated Pb^2+^ and filling V_*x*_). Effective passivation lowers the Urbach energy, increases PL, and suppresses Pb^0^ formation.
Redox shuttle: A reversible redox couple in the film (e.g., I^–^/I_2_/I_3_^–^ or Eu^3+^/Eu^2+^) that cycles between two oxidation states to oxidize Pb^0^ back to Pb^2+^ and reduce neutral iodine I_2_ to I^–^ at separated sites. Depending on the conditions, this can either limit Pb^0^ buildup or sustain iodine-driven degradation.
Precursor solution: Solution of AX, PbX_2_ salts, and additives in solvents forming coordination adducts that dictate nucleation, stoichiometry, and halogen availability. Ink chemistry strongly impacts defect densities and the tendency to form Pb^0^.
Perovskite composition: The choice of A-site, B-site, and halogen (ABX_3_ and derivatives, including mixed-cation/halide or low-dimensional phases) that sets defect formation energies, ion-migration pathways, and stability. For example, I-rich systems are generally more prone to Pb^0^ formation than Br-rich analogues.
